# Survey of the Extent of the Persisting Effects of Methylmercury Pollution on the Inhabitants around the Shiranui Sea, Japan

**DOI:** 10.3390/toxics6030039

**Published:** 2018-07-20

**Authors:** Shigeru Takaoka, Tadashi Fujino, Yoshinobu Kawakami, Shin-ichi Shigeoka, Takashi Yorifuji

**Affiliations:** 1 Kyoritsu Neurology and Rehabilitation Clinic, 2-2-28 Sakurai-cho, Minamata 867-0045, Japan; 2 Kikuyou Hospital, 5587 Haramizu, Kikuyou 869-1102, Japan; tds-fujino@jcom.zaq.ne.jp; 3 Minamata Kyoritsu Hospital, 2-2-12 Sakurai-cho, Minamata 867-0045, Japan; mkkawa@fsinet.or.jp (Y.K.); shigeoka@mk-kyouritu.com (S.-i.S.); 4 Department of Human Ecology, Graduate School of Environmental and Life Science, Okayama University, 3-1-1 Tsushima-naka, Kita-ku, Okayama 700-8530, Japan; yorichan@md.okayama-u.ac.jp

**Keywords:** methylmercury, long term exposure, symptoms, neurological findings, severity, delayed toxicity, correlation of signs and symptoms, dose-response relationship

## Abstract

In 1956 methylmercury poisoning, known as Minamata disease, was discovered among the inhabitants around the Shiranui Sea, Kyushu, Japan. Although about five hundred thousand people living in the area had supposedly been exposed to methylmercury, administrative agencies and research institutes had not performed any subsequent large scale, continuous health examination, so the actual extent of the negative health effects was not clearly documented. In 2009, we performed health surveys in order to examine residents in the polluted area and to research the extent of the polluted area and period of pollution. We analyzed data collected on 973 people (age = 62.3 ± 11.7) who had lived in the polluted area and had eaten the fish there and a control group, consisting of 142 persons (age = 62.0 ± 10.5), most of whom had not lived in the polluted area. Symptoms and neurological signs were statistically more prevalent in the four groups than in the control group and were more prevalent and severe in those who had eaten most fish. The patterns of positive findings of symptoms and neurological findings in the four groups were similar. Our data indicates that Minamata disease had spread outside of the central area and could still be observed recently, almost 50 years after the Chisso Company’s factory had halted the dumping of mercury polluted waste water back in 1968.

## 1. Introduction

In 1956, methylmercury poisoning was discovered among the inhabitants around Minamata Bay of Shiranui Sea in Kumamoto Prefecture, Kyushu, Japan. The condition, which was caused by the ingestion of fish and shellfish that had been contaminated by methylmercury, became known as Minamata disease [1]. For 36 years, from 1932 to 1968, the Nihon Chisso Company’s Minamata factory, which produced acetaldehyde, had been discharging waste water contaminated with methylmercury, created during the process, into the Shiranui Sea. Although it was suspected that about five hundred thousand people living in the area had been exposed to methylmercury poisoning, there have subsequently been very few comprehensive health surveys and examinations.

Certification of Minamata disease (methylmercury poisoning by eating fish) has been determined by the Pollution-Related Health Damage Compensation Law (PHDCL). Those victims must themselves personally apply to the Judgment Committee for Minamata Disease Accreditation (an advisory body to the Governor of the Kumamoto Prefecture) to be certified. According to the 1977 Diagnostic Criteria of Minamata disease, patients must have lived in the designated area at least one year before 1968, and four-limb somatosensory disturbance must be accompanied by at least one of the more severe symptoms of Hunter Russell syndrome such as ataxia, visual field constriction, and so on [2].

Actual judgment is stricter. A report by a neurologists in 1997 suggested that patients with such plural symptoms of Hunter Russell syndrome had been rejected by the Judgment Committee [3].

**Figure 1:**
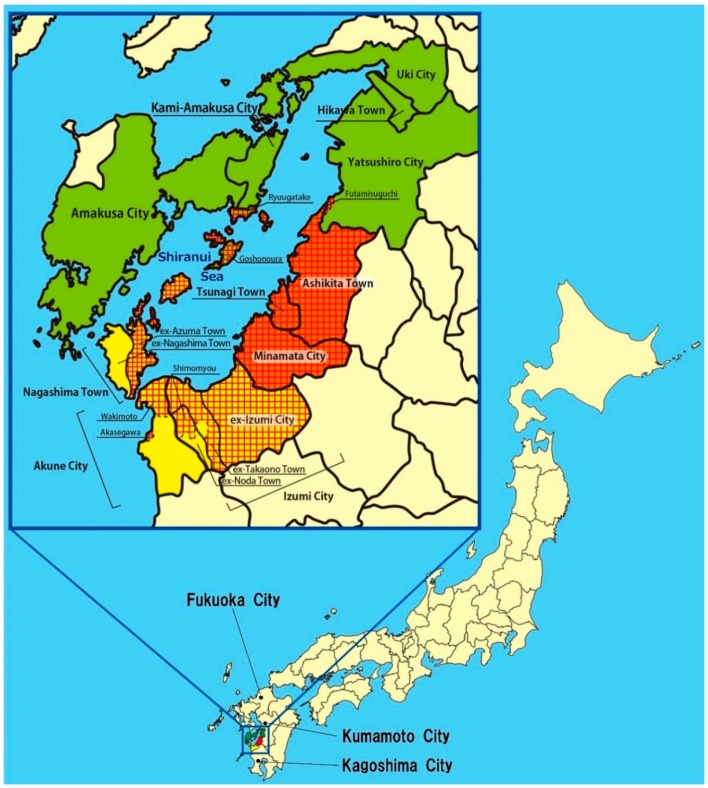
Surveyed area and designated area. Minamata Area: red, Northern Area: green, Southern Area: yellow, Other Areas: beige. Designated Area: red cross-hatched pattern. Control area: around Fukuoka, Kumamoto, and Kagoshima City.

The designated area has not been determined by epidemiological study but restricted to the place where severe Minamata disease victims have been discovered since the outbreak. Therefore, strangely enough, there is an enclave (Akasegawa district) in Akune City (Figure 1). Originally, the designated area was restricted to Minamata City, Tsunagi Town, Ashikita Town (including ex-Tanoura Town), and ex-Izumi City area by the PHDCL in 1971. However, it later became clear that the effects were more outspread and the extent of the area was expanded.

As of 2009, when time-limited special relief law (The Law Concerning Special Measures for the Relief of Minamata Disease Victims and the Settlement of Minamata Disease Issues, LSRS) was enforced, the designated area was restricted to the red cross-hatched areas in Figure 1, which consists of Minamata City, Tsunagi Town, Ashikita Town, Goshonoura of Amakusa City, Ryuugatake of Kami-Amakusa City, Futamisuguchi of Yatsushiro City in Kumamoto Prefecture and Izumi City, ex-Azuma Town area of Nagashima Town, Wakimoto and Akasegawa of Akune City in Kagoshima Prefecture (Table 1). Strictly speaking, the area designated by the PHDCL and that by LSRS are different. The designated area we refer to in this paper is the one stipulated by the Special Law (LSRS) that was in force between 2009 and 31 July 2012.

As of 2007, only 2268 patients were accredited by the administration [4] as suffering from Minamata disease, despite the fact that more than 17,000 people had applied for Minamata disease certification prior to 1999 [5]. In 1995, 8831 individuals were partially compensated for health problems, which included somatosensory disturbance [4], but they were not certified as actually having Minamata disease. Applicants seeking recognition were being socially discriminated against and the lack of a comprehensive pollution survey meant that many residents with health problems had not sought diagnosis.

However, in October 2004, after the Supreme Court of Japan ruled that the criteria stipulated by the Japanese Environmental Protection Agency for Minamata disease accreditation was too strict, an increasing number of residents applied for examination, therapy, and treatment for methylmercury poisoning. By the summer of 2009 over thirty thousand had applied and by the end of 2012 the number had risen to sixty thousand. In order to study the signs and symptoms of residents who hoped to be certified, we carried out a survey in September 2009 to determine the geographical extent and chronological development of methylmercury poisoning in the area.

From November 2004 to August 2009, we had already examined 3800 residents in the polluted area and found a lot of patients with neurological signs and symptoms. We performed this survey in order to research the prevalence of signs and symptoms as well as the geometrical and chronological spread of health problems caused by methylmercury.

In this study, we investigated not only the state of health (symptoms and neurological signs) in methylmercury-exposed people at present but the extent and mutual relationship of exposure level, onset and course of symptoms, severities of signs and symptoms and the significance of the designated area in the polluted region.

Also, the damage to health from methylmercury poisoning has been so great in Japan that researchers have concentrated on finding severe neurological abnormalities and have ignored milder health disturbances. Therefore, we also analyzed the data from subjects in whom sensory disturbances had not been detected during their physical examination.

## 2. Materials and Methods

### 2.1. Subjects

The study, which was planned for people who had lived in the methylmercury-polluted area and hoped to be certified for Minamata disease, was carried out on 20 and 21 September 2009. Information regarding the study was spread through media such as television, newspapers, and local government pamphlets. We received applications from people living throughout the coastal regions of the Shiranui Sea. Of the 1700 applicants we selected the first 1420 and informed them of the times, dates and places for the examinations.

Examinations were conducted at twelve sites in Kumamoto Prefecture and at five sites in Kagoshima Prefecture. A total of 1044 subjects were examined. They were given both written and verbal information about the examination method, how the data would be used and that their confidentiality would be protected. Of the 1044 subjects examined 974 residents agreed to having their data analyzed. The Ethics Committee of Minamata Kyoritsu Hospital approved the implement and analyses of this study. Among 974 subjects, one subject had not lived in the polluted area, so we excluded this subject. Finally, we analyzed 973 residents (M/F = 482/491, age = 62.3 ± 11.7, range = 33–92).

We grouped the exposed subjects into four categories, according to their living places at the time of the examination. Subjects who lived in Minamata City or Ashikita County (Tsunagi Town and Tanoura Town) at the examination time were classified as Minamata Area (*n* = 259, M/F = 136/123, Age = 62.5 ± 13.6). Subjects who lived in the northern area including Amakusa City, Kami-Amakusa City, Yatsushiro City, Yatsushiro County (Hikawa Town), or Uki City were classified as Northern Area (*n* = 279, M/F = 147/132, Age = 64.0 ± 11.1). Subjects who lived in Izumi City, Izumi County (Nagashima Town), or Akune City were classified as Southern Area (*n* = 246, M/F = 121/125, Age = 63.0 ± 11.1). Subjects who lived in the polluted area from 1950s to 1970s and were now living in other areas of Japan at the time of the examination were classified as Other Area (*n* = 189, M/F = 88/101, Age = 58.6 ± 9.9) (Table 1 and Table 2, Figure 1).

The Control group was comprised of 227 persons chosen from hospital staff and residents living around Fukuoka City, Kumamoto City, and Kagoshima City in 2006 and 2007. In this control group, younger subjects were over-represented, and were excluded. Finally, we selected 142 residents (*n* = 142, M/F = 56/86, age = 62.0 ± 10.5, range = 36–86). Most of them had worked in the service sector. The subjects in the control group were the same as those mentioned in another paper in 2008 [6].

### 2.2. Epidemiological Conditions

Due to the absence of a comprehensive pollution survey in the Minamata area, most of the residents have not undergone any tests to determine the mercury levels in their bodies resulting from their exposure to mercury and methylmercury. Therefore, we prepared a questionnaire to collect information about their places of residence, dietary habits, occupations and family health. Such information is important and useful, because, although a large part of the local diet consists of fish, there have been no previous surveys done to gather accurate information. All subjects had eaten fish and shellfish from the Shiranui Sea. In order to assess the indirect methylmercury exposure, when subjects were asked about the frequency of fish ingestion they were asked to choose from one of the five answers (3 times/day, 2 times/day, once/day, more than once/week, less than once/week).

According to their residential history, subjects were classified into one of the following three categories. The first category consisted of subjects who had lived in the designated area for at least one year (DA: *n* = 786, M/F = 386/400, Age = 63.0 ± 11.3). The second category consisted of subjects who had not lived in the designated area for at least one year (NDA: *n* = 158, M/F = 85/72, Age = 63.6 ± 10.0). Those who had been born or had moved to the polluted on or after 1 January 1969 were classified under the third category (BA1968: *n* = 30, M/F = 21/9, Age = 37.4 ± 2.3).

In order to analyze the younger generation, we re-analyzed 30 subjects who were born after 31 December 1968. To evaluate this group, we selected 88 out of 227 subjects whose age was lower than 49 from the Control Area (M/F = 40/48, Age = 37.5 ± 6.0), and 84 out of 786 exposed subjects in the designated area who were born after 31 December 1968 (M/F = 44/40, Age = 44.8 ± 2.3) and whose age was lower than 49 from the four exposed groups.

The subjects’ age, sex, smoking and alcohol drinking habits, frequency of fish intake, the occupation of the subjects and the subjects’ parents, complications, history of application for Minamata disease, family history of Minamata disease, and the witnessing of abnormal animal behavior was analyzed for each group.

### 2.3. Onset of Symptoms

Subjects from the four polluted areas were asked about the time of onset of their first abnormal experience supposed to be related to methylmercury exposure, muscle cramps, four-limb numbness, stumbling tendency, difficulty in fine finger tasks, and limited peripheral vision. The time between the appearance of the first symptom until all five symptoms (cramps, numbness, stumbling tendency, difficulty in fine finger tasks, and limited vision) had appeared were calculated. When a subject could not answer with certainty, the approximate year or median of the generation was used.

The latency period between methylmercury exposure and onset of health problems can be roughly estimated by this information.

### 2.4. Questionnaire on Complaints

Our questionnaire consisted of 57 questions. Seven questions were related to sensory impairment, 6 related to somatic pain, 6 related to visual impairment, 3 related to hearing impairment, 3 related to tasting and smelling problems, 9 related to in-coordination of the extremities, 5 related to other movement impairment, 4 related to vertigo and dizziness, 3 related to general complaints, 11 related to emotional and intellectual problems. The subjects were asked to answer each question by selecting from one of the following four possibilities: (1) Yes, always; (2) Yes, sometimes; (3) Yes in the past but No at present; (4) Never. The prevalence of each complaint was calculated for each group and compared. The subjects completed the questionnaire before they were examined. Any subjects who were unable to complete the questionnaire by themselves were questioned orally. The results of the questionnaires were reviewed prior to the examination.

### 2.5. Neurological Examination

A neurological examination was performed on all subjects. The examination comprised the following tests: dysarthria, auditory disturbance, visual constriction, gait disturbance, tandem gait, Mann’s test, balancing on one foot (eyes open and closed), finger-to-nose test (eyes open and closed), diadochokinesis, heel-to-knee test, postural tremor of hand, and superficial sensory disturbance (touch and pain).

Dysarthria, auditory disturbance, visual constriction, and postural tremor were judged as present or absent. Dysarthria, auditory disturbance, and visual field were judged by the examining physician without using special instruments. Visual disturbance was considered present when the subject had a lateral visual field of 80 degrees or less, as measured by the confrontation visual field test.

Limb and truncal ataxia were judged as absent, mildly abnormal, or distinctly abnormal. Tandem gait disturbance was judged as distinctly abnormal if the subject could not walk more than 5 steps and as mildly abnormal if they could walk 5 steps but were unstable. The one-foot standing and Mann’s test were judged as distinctly abnormal if it was impossible for the subject to keep their balance for more than 3 s and as mildly abnormal if they could keep their balance for more than 3 s. Finger-to-nose test and heel-to-knee test were judged as distinctly abnormal if there was constant dysmetria or decomposition and as mildly abnormal if there was uncertain dysmetria, decomposition, or slow progress to the destination. Dysdiadochokinesis was judged as distinctly abnormal if there was a constant abnormality and as mildly abnormal if there was an uncertain abnormality or slow movement.

For sensory disturbance, tactile sense was examined by comparing chest area and dorsal side of both hands and both foot by using a calligraphy brush. After that, truncal and perioral tactile sense was examined by touching the skin softly with the brush. Pain sense was examined by comparing chest area and dorsal side of both hands and both foot by using a 20 g needle for pain inspection. After that truncal and perioral pain sense were examined by the same needle. The needle was attached to a 20 g handle. We evaluated the tactile and pain sense both by relative evaluation between different sites of bodies and by absolute evaluation, especially for pain. When general tactile sensory disturbance was suspected, a physician asked of the subject to close his or her eyes and indicate which part of their body was being touched by the calligraphy brush.

One-hundred and forty-four doctors, including neurological specialists, carried out primary and secondary examinations on the subjects. The sensory examination was repeated by more trained physicians on all subjects.

All the physicians participating in the study were trained in the procedures by direct instruction, written instruction or visual instruction in the form of video tutorials. The physicians who performed the neurological examination in the control areas were different from those in the polluted area, but the methods and criteria of the neurological examination was the same as those preformed in the polluted areas.

### 2.6. Statistical Methods

As formerly mentioned in Section 2.1 in this chapter, we classified the methylmercury-exposed subjects into four groups: Minamata Area, Northern Area, Southern Area, and Other Areas. We compared them with the Control Area. All the calculations were performed using MS Excel 2010 and STATA ver.14. The prevalence of data was analyzed by using MS Excel and STATA. Logistic regression analysis and correlation analysis were performed by STATA. When the prevalence of the control group was zero in an item of symptoms or signs, we postulated that the eldest subject in the control group was positive and calculated the OR and confidence interval by STATA. The analysis of the onset year was done in MS Excel.

#### 2.6.1. Questionnaire on Symptoms and Neurological Examination

The data percentages of the answers “always yes” and “always or sometimes yes” from the questionnaire were summed, the results analyzed and the correlations between the control and the four exposed groups were calculated. A total of 23 questions out of the 57 questions asked were analyzed. Three of the 7 questions relating to sensory impairment, 2 of the 6 relating to somatic pain, 1 of the 6 relating to visual impairment, 1 of the 3 relating to hearing impairment, 2 of the 3 relating to tasting and smelling problems, 5 of the 9 relating to in-coordination of the extremities, 2 of the 5 relating to other movement impairment, 1 of the 4 relating to vertigo and dizziness, 1 of the 3 relating to general complaints, and 5 of the 11 relating to emotional and intellectual problems were selected and analyzed.

The prevalence of each sign and symptom from the five groups (1 control and 4 exposed groups) were calculated. Odd’s ratio for the association between area and symptoms or signs were calculated by logistic regression analysis. Correlation of prevalence among the control group and the four other exposed groups were calculated for symptoms (always “yes”), symptoms (always or sometimes “yes”), and neurological signs.

#### 2.6.2. Score for Symptoms (Always), Symptoms (Always and Sometimes), and Neurological Signs

To evaluate the severity of the neurological signs, we added (a) mark(s) to positive signs and symptoms, and we calculated the total score in the exposed four groups.

As to the symptom score (always), we added 1 point when a subject’s answer was “always”. Symptoms score (always) ranged from 0 to 23. As to symptoms (always and sometimes), we added 2 points when a subject’s answer was “always”, and added 1 point when a subject’s answer was “sometimes”. Symptoms score (always and sometimes) ranged from 0 to 46. A score of zero was given to “no answer” items.

As to the neurological signs, the cranial nerve score (6 points) consists of dysarthria (2), auditory disturbance (2), visual constriction (2). The upper, lower ataxia and tremor score (5 points) consists of finger-to-nose test (eyes open) (1), finger-to-nose test (eyes closed) (1), diadochokinesis (1), heel-to-knee test (1), and postural tremor (1). The truncal ataxia score (5 points) consists of normal gait disturbance (1), tandem gait disturbance (1), Mann’s test (1), balancing on one foot (eyes open) (1), and balancing on one foot (eyes closed) (1). To evaluate upper, lower, and truncal ataxia, 1 point was given for both mild and distinct abnormalities. The sensory score (6 points) consists of four-limb peripheral touch disturbance (1), perioral touch disturbance (1), systemic touch disturbance below the neck (1), four-limb peripheral pain disturbance (1), perioral pain disturbance (1), systemic pain disturbance below the neck (1). We finally totaled the four scores (22 points) for cranial nerve (6), upper, lower ataxia and tremor (5), truncal ataxia (5), and sensory (6). A score of zero was given to the “no data” item.

The scores were calculated for each area. To research the presence of dose-response relationship, each score according to the frequency of fish ingestion in the exposed groups were calculated.

#### 2.6.3. Analyses among Fish Ingestion, Score of Signs and Symptoms, and the Onset of Symptoms

In Minamata, most of the residents had not had their mercury levels measured during the most polluted period or in the subsequent period when mercury level had decreased. Instead of such direct mercury pollution values, we used frequency of fish ingestion as an indirect indication of methylmercury exposure.

To estimate dose-response (from methylmercury exposure to health effects) relationships, we calculated scores of signs and symptoms for each frequency of fish ingestion. In order to estimate the difference of latency period from exposure of mercury by exposure levels, we calculated the average year of onset and the average interval between the first symptom and the onset of each following symptom in each frequency of fish ingestion.

Lastly, we evaluated the correlation between the score for signs and symptoms, and onset period of symptoms to estimate difference of latency period and severity of methylmercury poisoning.

#### 2.6.4. Characteristics of Signs and Symptoms in Subjects Whose Sensory Disturbance Was Not Recognized

To estimate the degree of health disturbance in subjects in whom sensory disturbance had not been detected during their physical examination, we re-classified the exposed subjects into two groups. Sensory disturbance (−) group (*n* = 91, M/F = 64/27, Age = 59.7 ± 13.3) consisted of subjects whose sensory score was zero, in whom neither four-limb peripheral disturbance, perioral disturbance, systemic disturbance below the neck in tactile or pain examination met the criteria required for recognition. Sensory disturbance (+) group consisted of other subjects whose sensory score was one to six (*n* = 882, M/F = 428/454, Age = 62.6 ± 11.6). The control group was the same as those used in the first analysis. We compared signs and symptoms between the three groups.

## 3. Results

### 3.1. The Subjects’ Background

The demographic characteristics of the subjects from the five groups are shown in Table 2. The age for Other Areas was significantly lower than other groups. Smoking and alcohol drinking was almost the same in the different groups. The frequency of fish intake was significantly higher in the exposed groups than the control. Hypertension, diabetes mellitus, and orthopedic diseases were significantly more prevalent in the polluted area. Subjects who had applied for Minamata disease certification were only 8.6% to 15.4%, even in the polluted area, although many (38.7–79.9%) of them had a family history of Minamata disease.

### 3.2. Questionnaire on Symptoms

**Figure 2:**
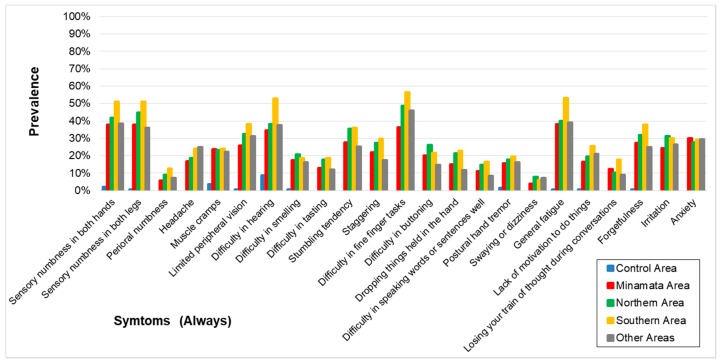
Prevalence of symptoms (Always) in each area. Prevalence was higher in the four exposed groups than that in the control area. The symptomatic patterns were similar among the four groups.

The symptoms (always) are shown in Table 3 and Figure 2. For most of the symptoms (always), Odds ratio (OR) for the association between area and signs or symptoms calculated by logistic regression analysis and adjusted for age, sex, diabetes mellitus, and orthopedic diseases were very high and the lower limits of the OR were higher than 1, except for two questions (“perioral numbness” and “swaying or dizziness” between control and Minamata Area). The correlation among the four exposed groups on the prevalence of symptoms (always) (Table 4) were extremely high (0.9246–0.9611) whereas the correlation between the control and the four exposed groups were very low (0.2751–0.3866).

**Figure 3:**
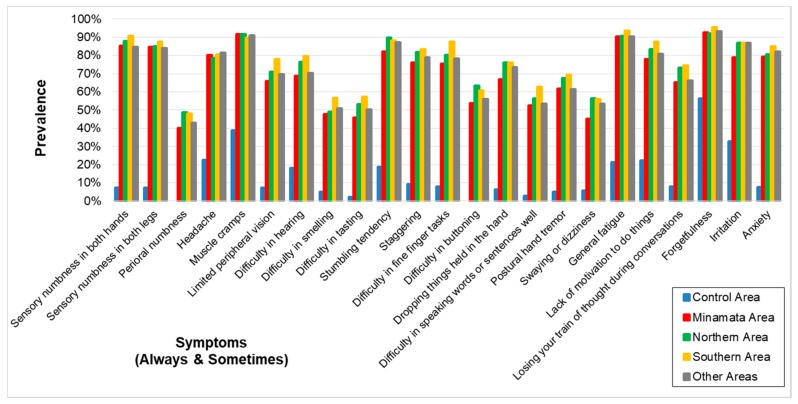
Prevalence of symptoms (Always and Sometimes) in each area. Prevalence was higher in the four exposed groups than that in the control area. The symptomatic patterns were similar among the four groups.

As to the symptoms (always or sometimes), the OR for the same analysis and adjustment were very high and the lower limits of the OR were all higher than 1 (Table 5, Figure 3). The correlation among the four exposed groups on the prevalence of symptoms (always and sometimes) (Table 6) were extremely high (0.9778–0.9875) whereas the correlation between the control and the four exposed groups were lower (0.6501–0.7046).

### 3.3. Neurological Signs

**Figure 4:**
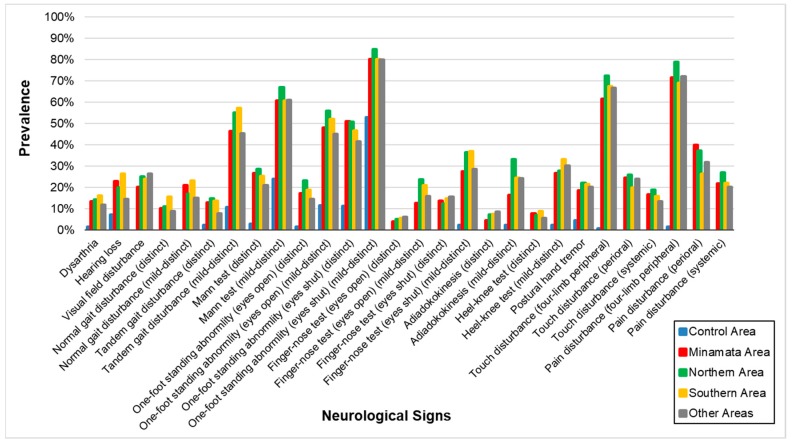
Prevalence of neurological signs in each area. Prevalence was higher in the four exposed groups than that in the control area. The patterns of signs were similar among the four groups.

The results of the neurological signs are shown in Table 7 and Figure 4. Almost all signs were higher in the exposed groups than the control except for the “finger-nose test (opening eyes) (distinct)” in the three polluted areas and “adiadokokinesis (distinct)” in the two polluted areas.

The correlation among the four exposed groups on the prevalence of neurological signs (Table 8) were extremely high (0.9716–0.9864), compared to the correlation between the control and the four exposed groups (0.6327–0.6802).

### 3.4. Scores of Signs and Symptoms

Scores of signs and symptoms were shown in Table 9. All scores were significantly higher and also similar in the four exposed groups. After recalculating the data in DA, NDA, and BA1968, even NDA and BA1968, scores were extremely high in the exposed groups (Table 10).

**Figure 5:**
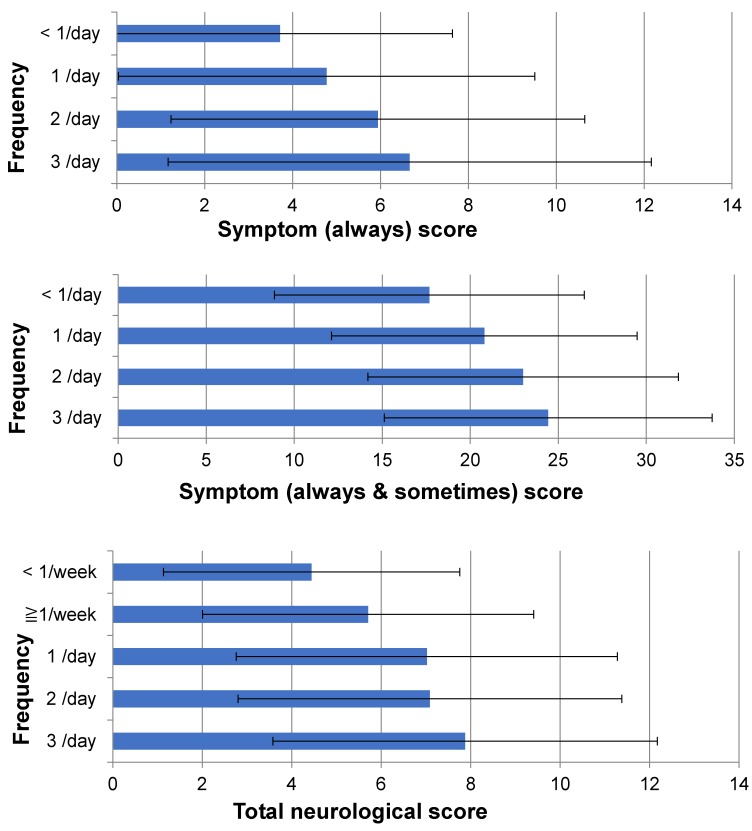
Scores and frequency of fish ingestion (*n* = 973). Scores increased as the frequency of fish ingestion increased.

Relations between scores and frequency of fish ingestion are shown in Table 11 and Figure 5. The symptom (always) score was higher when the frequency of fish ingestion was more. Although scores in the “<1/week” group were higher than in the “1/day” and “≥1/week” groups, the causes were unknown, so we merged the scores in “≥1/week” and “<1/week” into “<1/day” in Figure 5.

The symptom (always and sometimes) score was higher when frequency of fish ingestion was more. As with the symptom (always) score, scores in the “<1/week” group were higher than those in the “≥1/week” group, the causes were unknown, so we merged the scores in “≥1/week” and “<1/week” into “<1/day” in Figure 5.

The total neurological score was higher when frequency of fish ingestion was more (Table 11, Figure 5).

**Figure 6:**
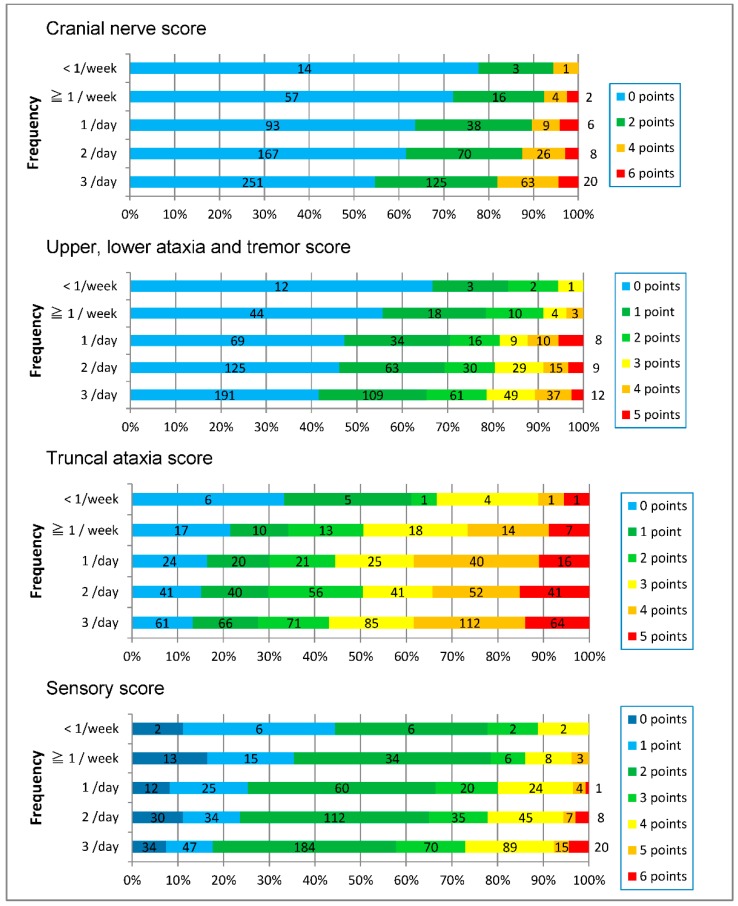
Each score increased as the frequency of fish ingestion increased.

In each of the four neurological scores (cranial nerve score, upper, lower ataxia and tremor score, truncal ataxia score, and sensory score), the higher the frequency of fish ingestion, the more the score increased. But the range of scores in each frequency group was greater between the zero to maximum score (Figure 6).

### 3.5. Onset of Symptoms

#### 3.5.1. Onset of Symptoms in Each Group

Table 12 shows onset year of symptoms in each group. Average year of the first symptom is 1979.0 ± 14.8 in the Minamata Area. That is more than 10 years after the Chisso Factory stopped releasing polluted waste-water in May 1968. Sixty-five percent (628/996) in the exposed groups displayed the first symptom later than 1968. The onset of the first symptom, muscle cramps, four-limb numbness, stumbling tendency, difficulty in fine finger task, and limited peripheral vision were slightly later in subjects in the Minamata Area, but almost the same among four exposed groups. In the Minamata Area, more residents had already been examined for methylmercury poisoning than in other groups, which might be related to the reason why the average year of onset was later than other places.

**Figure 7:**
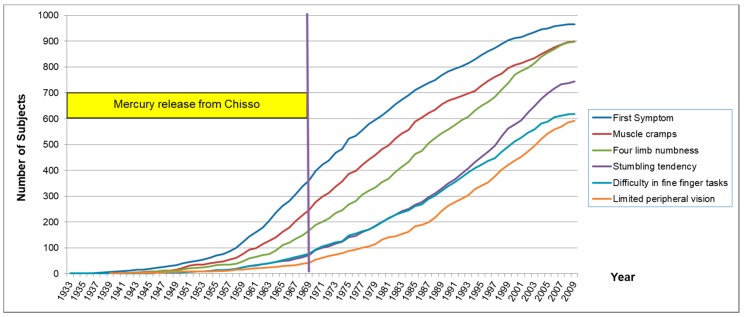
Onset year of each neurological symptom (actual number). Sixty-five percent of the subjects in the exposed area experienced their first symptoms after 1968, when polluted waste water from the Chisso Company’s factory was halted. Symptoms have even appeared in recent years.

Muscle cramps occurred 4.8 ± 8.9 years after the first symptom. Four-limb numbness (8.7 ± 11.8 years), difficulty in fine finger task (13.2 ± 12.7 years), stumbling tendency (14.3 ± 13.5 years), and limited peripheral vision (17.3 ± 13.3 years) were followed. Figure 7 shows the total number of subjects with each symptom.

**Figure 8:**
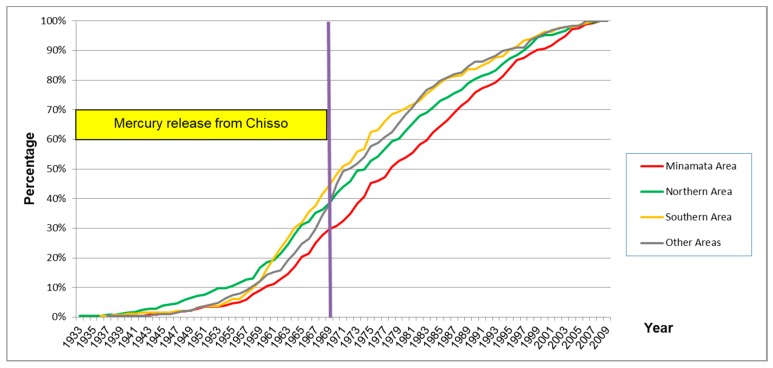
Onset year of the first symptom in each exposed group (percentage). Cumulative curves of the onset were almost the same among the four exposed groups.

The appearance of symptoms was almost the same among the four exposed groups. Figure 8 shows the cumulative rate of onset year of the first symptom in each exposed group.

#### 3.5.2. Relation between the Onset of Symptoms and Frequency of Fish Ingestion

**Figure 9:**
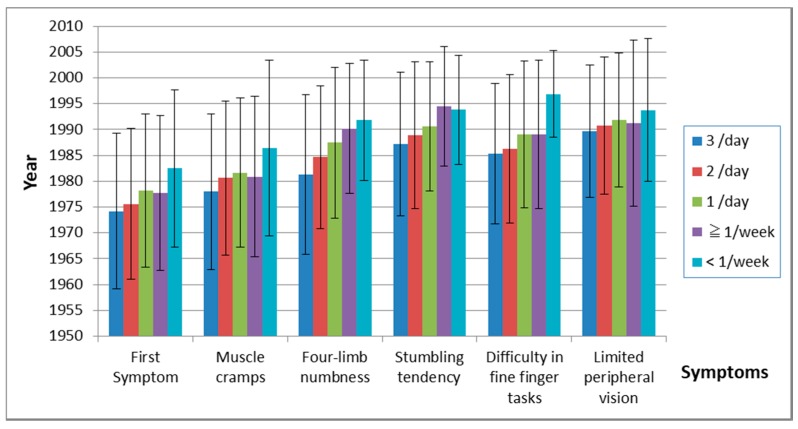
Onset of symptoms and frequency of fish ingestion. The time of onset of subsequent symptoms increased as the frequency of fish ingestion decreased.

**Figure 10:**
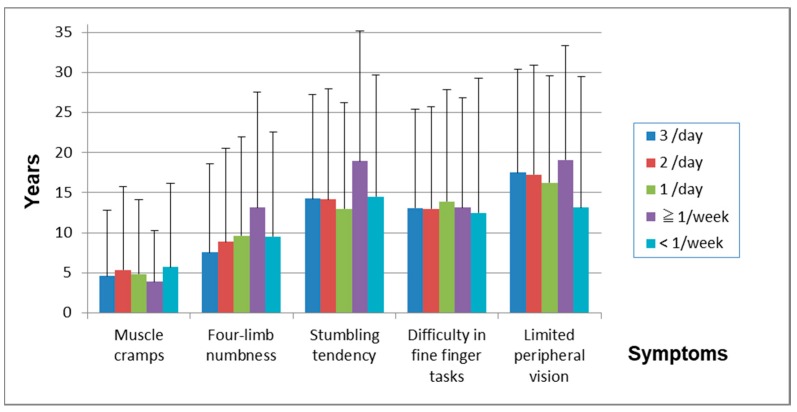
Years of each symptom after the first symptom. There was almost no relation between fish ingestion and duration from the first symptom to each following symptom, except for four-limb numbness.

The frequency of fish ingestion was closely related to the onset year of other symptoms (Table 13, Figure 9). Figure 10 shows duration between the first symptom and each other symptom. The duration between the first symptom and four-limb numbness was related to the frequency of ingestion, but there were almost no relations in other symptoms.

#### 3.5.3. Relations between Onset of Symptom and Scores of Signs and Symptoms

The onset of symptoms was related to scores. When the onset year was earlier, the score became greater (Table 14).

### 3.6. Health Effect in Younger Generation

#### 3.6.1. Characteristics of Younger Subjects in the Exposed and the Control Area 

The demographic characteristics of subjects in younger subjects are shown in Table 15. Many of the younger subjects who were born later than 1968 (BA1968) in the exposed areas had also lived in fishermen families (30.0%) and had eaten more fish (3 times/day in 50.0%) than the elderly exposed subjects. While the prevalence of subjects whose parents were fishermen and the prevalence of subjects who had a family history of Minamata disease were almost the same between BA1968 (*n* = 30, in Table 15) and Designated Area (*n* = 84, in Table 15), there was no significant difference in complications between BA1968 and the Control Area (*n* = 88, in Table 15).

#### 3.6.2. Symptoms and Neurological Signs in the Younger Generation

**Figure 11:**
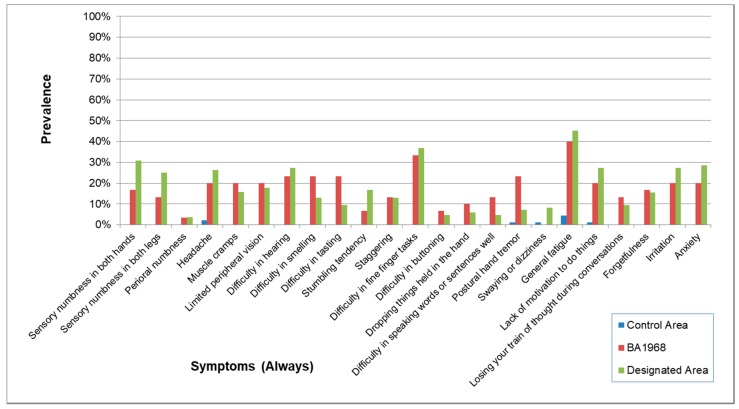
Prevalence of symptoms (Always) in the younger generation. Prevalence in BA1968 and the Designated Area were higher than in the Control Area. The symptomatic patterns in BA1968 and the Designated Area were similar.

**Figure 12:**
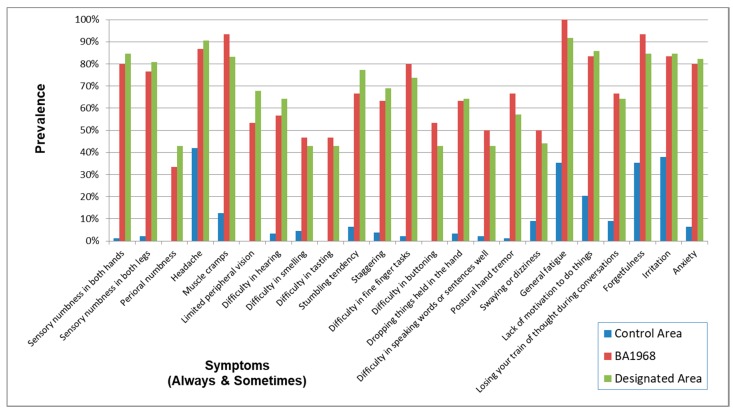
Prevalence of symptoms (Always and Sometimes) in the younger generation. Prevalence in BA1968 and the Designated Area were higher than in the Control Area. The symptomatic patterns in BA1968 and the Designated Area were similar.

**Figure 13:**
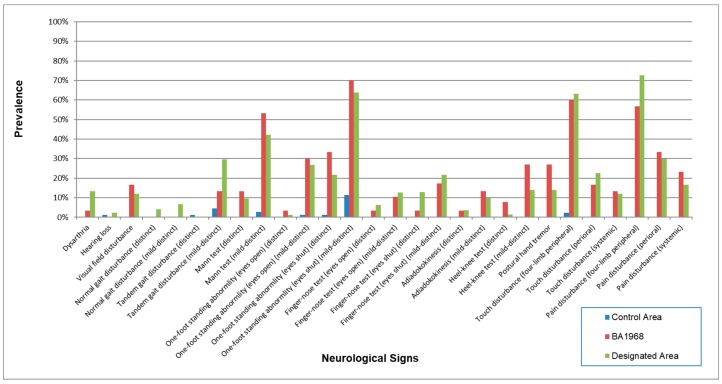
Prevalence of neurological signs in the younger generation. Prevalence in BA1968 and the Designated Area were higher than in the Control Area. The patterns of signs in BA1968 and the Designated Area were similar.

Symptoms and neurological signs in younger age (BA1968) were much more prevalent than in the Control Area, and the pattern of positive findings resembled those in the Designated Area (Figure 11, Figure 12 and Figure 13). The score of signs and symptoms showed similar results (Table 16).

#### 3.6.3. Onset of Symptoms of Designated, Non-Designated Area and Subjects Born after 1968

**Figure 14:**
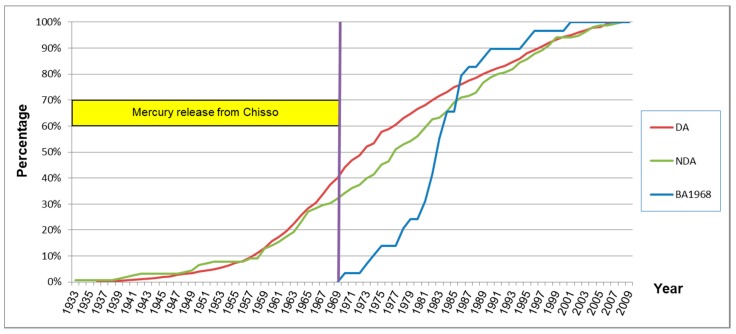
Onset year of the first symptom of subjects with and without residential history in the designated area (percentage).

Figure 14 shows the onset of the first symptom in the designated area (DA), non-designated area (NDA) and subjects born after the end of 1968 (BA1968). The classifications for these groups were the same as those in Table 10 (not in Table 15). The cumulative curves were almost the same between subjects who had lived in the designated and non-designated area. 82.8% (24/30) of the subjects who were born after 1968 had developed their first symptom by 1987.

### 3.7. Signs and Symptoms in Subjects in Whom Sensory Disturbance had not been Detected during Their Physical Examination 

**Figure 15:**
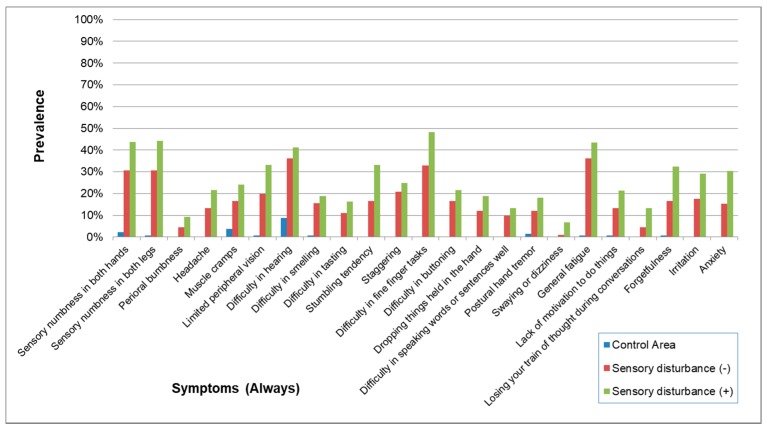
Prevalence of symptoms (Always) in subjects with and without sensory disturbance. The prevalence of symptoms was a little lower in subjects without sensory disturbance than in subjects with sensory disturbance but was apparently higher than the Control Area. The prevalence patterns were similar in exposed subjects with and without sensory disturbance.

**Figure 16:**
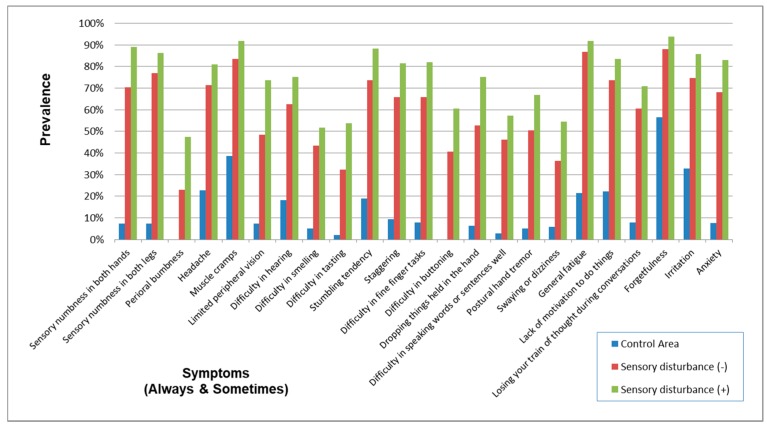
Prevalence of symptoms (Always and Sometimes) in subjects with and without sensory disturbance. The prevalence of symptoms was a little lower in subjects without sensory disturbance than in subjects with sensory disturbance but was apparently higher than the Control Area. The prevalence patterns were similar in exposed subjects with and without sensory disturbance.

**Figure 17:**
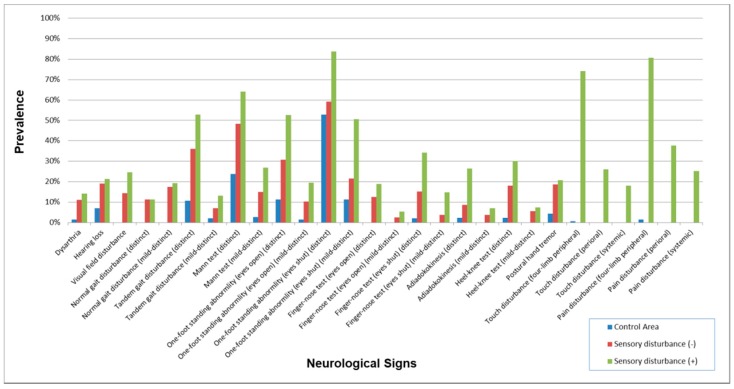
Prevalence of neurological signs in subjects with and without sensory disturbance. Except for the prevalence of sensory disturbance, that of other symptoms was lower in subjects without sensory disturbance than in subjects with sensory disturbance but was generally higher than the Control Area. The prevalence patterns were similar in exposed subjects with and without sensory disturbance except for the prevalence of sensory disturbance.

The demographic characteristics of subjects with and without sensory disturbance are shown in Table 17. Overall, the prevalence of symptoms (Always) and symptoms (Always and Sometimes) without sensory disturbance were a little lower than the subjects with some sort of sensory disturbance (Figure 15 and Figure 16). However, most of their symptoms were more prevalent than the control group. Furthermore, the prevalence pattern of neurological signs other than sensory disturbance in subjects without sensory disturbance was similar to that in subjects with some sort of sensory disturbance (Figure 17).

The scores of signs and symptoms in subjects without sensory disturbance were lower than those in subjects in the exposed area and higher than those in the control area (Table 18).

## 4. Discussion

### 4.1. Characteristics of the Subjects

The data collected on the occupations and dietary habits of the four methylmercury-exposed groups showed clearly that the majority are connected to the fishing industry and that their diets consist of large amounts of fish and shellfish.

Certified Minamata disease patients and compensated patients had lived in the designated area, which consisted of the whole Minamata Area, a large part of the Southern Area, and a small part of the Northern Area. In these areas, thousands of residents had already been examined for Minamata disease. But in the non-designated area, which included a part of the Northern and Southern Areas, as well as some other smaller areas, residents had not received information about methylmercury poisoning and had had little opportunity to be examined for methylmercury poisoning. These situations were reflected in the higher percentage of subjects (41.6%) who had not lived in the designated area in the Northern Area than the other three exposed groups (Table 1).

To adjust the higher prevalence of diabetes mellitus and orthopedic diseases in the exposed groups, we used logistic regression analysis. After adjustment for age, sex, diabetes mellitus, and orthopedic diseases, the prevalence of most signs and symptoms were extremely higher in the four exposed groups.

This means that neither diabetes mellitus nor orthopedic diseases were the cause of these signs and symptoms. However, on the contrary, methylmercury exposure might increase diabetes mellitus and orthopedic diseases. Shigenaga reported that in infantile and acute adult Minamata disease cases, injury to the pancreatic islet cells occurred [7]. Harada reported that deformity of four limbs (19%) and four-limb pain (36%) were observed in 145 family members of certified Minamata disease patients [8].

### 4.2. Characteristics of Signs and Symptoms of Chronic Methylmercury Poisonings

In this study, symptoms of methylmercury-intoxicated subjects varied widely. In 1973, Tatetsu et al. reported symptoms in 215 patients whom they diagnosed with Minamata disease and examined precisely using a two-point scale (“yes” or “no”). The symptoms (prevalence) included numbness of hands and feet (82%), dysesthesia of hands and feet (50%), pain in the head, back, lower back, four limbs (79%), fatigue (65%), visual disturbance (55%), limited peripheral vision (31%), difficulty in hearing (60%), difficulty in smelling (28%), difficulty in tasting (22%), stumbling tendency (58%), difficulty in fine finger tasks (37%), difficulty in buttoning (34%), difficulty in speaking (41%), muscle cramps (71%), hand tremor (43%), insomnia (43%), and forgetfulness (75%) [9].

From 1974 to 1979, Fujino used a two-point scale questionnaire to interview adult residents of Katsurajima Island, Izumi City and reported general fatigue (100%), forgetfulness, difficulty in calculation, inability to concentrate (95%), numbness (95%), difficulty in hearing (90%), muscle cramps (90%), insomnia (90%), staggering or stumbling (93%), difficulty in buttoning (88%), hand tremor (71%), limited peripheral vision (78%), difficulty in smelling (68%) in 41 adult residents [10]. The lower the age of the Katsurajima Island residents, the milder the symptoms.

In 1985, Kinjo et al. used a two-point scale questionnaire to interview certified Minamata disease patients in these designated areas. In Kinjo’s study, constriction of visual field (19.3%), difficulty in hearing (54.0%), difficulty in speaking (38.2%), difficulty in buttoning (52.8%), stumbling (69.3%), tremor (39.5%), hypoesthesia of the limbs (67.1%), dysesthesia of the limbs (88.4%), hypoesthesia of the mouth (25.4%), forgetfulness (88.4%), fatigue (82.9%), cramps (80.0%) were observed [11]. 24 years later the variety of symptoms was the same in non-certified residents as had been observed earlier in the certified patients.

In 2005, we performed a study of residents who had been exposed to methylmercury. The symptoms in the group without neurological complications (Always and Sometimes) were numbness of hands and feet (89%), limited peripheral vision (66%), difficulty in hearing (61%), stumbling tendency (63%), difficulty in buttoning (54%), muscle cramps (97%), hand tremor (75%), fatigue (88%), and forgetfulness (97%) [6].

**Figure 18:**
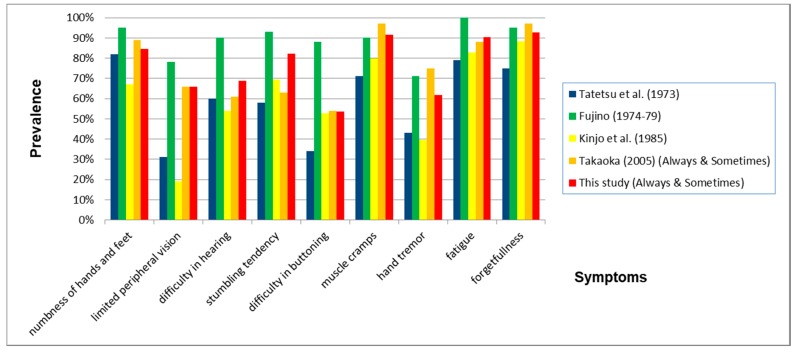
Prevalence of symptoms when comparing nine questions common to all five studies. The prevalence of all characteristic symptoms for Minamata disease was very. In this study, we used the prevalence of “sensory numbness in both hands” instead of “numbness of hands and feet”.

The prevalence pattern of symptoms (Always and Sometimes) were similar to questions common to three of the earlier studies—Tatetsu et al., Kinjo et al. and Takaoka (2005), as well as the present Takaoka study. Fujino’s study showed much more severe symptoms than the four previously mentioned studies (Figure 18). The similarities in symptom patterns may be due to the fact that the effects of methylmercury poisoning on health have persisted into the twenty-first century. Because there were differences in these studies in the selection of subjects, phrasing of questions, and the scoring methods’ choice of answers were not necessary the same, the percentages of each study, shown below, do not necessarily represent the severity of subjects’ symptoms.

Sugiura studied fishermen in Minamata City (1990) and in Izumi City (1988) and pointed out that health problems were present not only in certified patients but also in uncertified patients and other fishermen [12]. In our study, there were no certified patients, but most of them had similar symptoms.

Although activities of daily living (ADL) of these patients decreased from 60 years old, 9.1% still needed assistance in eating, 11.6% in bodily hygiene and 10.6% in using the toilet [11]. The ADL level of most subjects in this study was “Independent” so they were able to come for the examination without serious assistance. The effects of chronic methylmercury cover a wide range, and disabilities remain or progress slowly in many cases.

The symptoms in the questionnaire consist of those both specific and non-specific to methylmercury poisoning. The symptoms whose percentage of the answer (always and sometimes) in the Control Area were considerably lower, compared to those in the exposed groups (e.g., “perioral numbness”, “difficulty in tasting”, “difficulty in buttoning”), are supposed to more specific symptoms. Table 2 and Table 4, Figure 2 and Figure 3 show that prevalence of specific symptoms as well as that of non-specific symptoms became higher through methylmercury exposure.

Increases in specific symptoms from methylmercury poisoning mean that those high percentages of symptoms include the effects of methylmercury. Also increases of non-specific symptoms in exposed people mean that methylmercury also has non-specific health effects.

The prevalence of symptoms was highly correlated among the four exposed groups. By the effects of non-specific symptoms, there were some correlations between the control and the four exposed groups, but the correlations were extremely high among the exposed groups (Table 3 and Table 4, Figure 2). These patterns of symptoms were supposed to reflect the characteristics of methylmercury poisonings.

That the correlation between the control and exposed groups in symptoms of “always and sometimes” became higher than that in symptoms of “always” can be explained by effects of the non-specific symptoms for methylmercury poisoning in the control area (Table 5 and Table 6, Figure 3).

As with symptoms, positive neurological signs in the four exposed groups were highly more prevalent than the control area (Table 7, Figure 4). Also, the correlation among the four exposed groups was also supposed to reflect the characteristics of methylmercury poisoning (Table 8). Furthermore, the concordance between these findings by 144 doctors with different specialties suggests that the instructions for the examinations were successful.

Many factors may have influence on the final prevalence including possible bias in the selection of subjects, variations in the doctors’ examination technique, judgment criteria, the subjects’ age, and other health complications. Despite the possible influence of these factors, the high level of consistency in the gathered results show that there was a high degree of precision involved. Also, the collected data suggests that the effects of methylmercury contamination are still present today.

Clinical signs were also as various as studies previously reported. According to Tatesu et al., 269 residents in Minamata district, diagnosed with Minamata disease in 1972, showed sensory disturbance (97.0%), ataxia (93.7%), hearing disturbance (84.0%), weakness (67.3%), speech disturbance (62.5%), and constriction of visual field (59.5%) [9].

Fujino, in his study from 1974 to 1979, reported that those who had lived longer on Katsurajima Island, showed a higher prevalence in all signs of Hunter Russell syndrome: somatosensory disturbance, ataxia, constriction of visual fields, auditory disturbance, and dysarthria. The combination of these symptoms became less as the subjects’ ages decreased [10]. In a study from 1975 to 1979, Ninomiya et al. reported that hypoesthesia (60.3%), ataxia (20.7%), impairment of hearing (43.8%), visual change (25.6%), and dysarthria (13.2%) were recognized in 121 residents from the polluted area in Goshonoura [13].

In our study from 2005, subjects in the methylmercury-polluted area had dysarthria (23%), auditory disturbance (33%), visual constriction (28%), positive finger-nose test (50%), heel-shin test (48%), normal gait disturbance (41%), and poor one-foot standing (66%) [6].

Because these studies differed in the selection of subjects, examination method, and judgement criteria, their positive neurological findings were not necessarily the same. However, common abnormalities in many functions including somatosensory, visual, and hearing acuities, coordination of upper and lower extremities were recognized, indicating that methylmercury poisoning still prevails.

### 4.3. Dose-Response Relationship of Chronic Methylmercury Poisoning

The severity of the subjects’ symptoms varied widely and some of them were experienced sporadically, which means that symptoms were persistent, intermittent, or periodical in many cases. The severity of neurological signs ranged from no finding to continual seriousness. The seriousness generally correlated with the volume of fish ingested (Table 11, Figure 5 and Figure 6), but even between subjects with a low intake of fish and those with a high intake the severity of the cases varied from no findings to findings with high severity were observed (Figure 6). This is the first study that dose-response effects were observed in methylmercury poisoning in Japan.

According to pathological studies on Minamata disease, a spongy state, from complete loss of neurons in the cerebral and cerebellar cortex, can occur in the severe cases, whereas in milder cases, “scattered single cell necrosis” in the cortex is supposed to occur [14]. Nowadays, this cellular loss in milder cases has been supposed to be caused by “apoptosis” [15]. This could explain the variability in seriousness of this disease.

A higher percentage of intermittent or periodical signs and symptoms has been supposed be characteristics of mild and moderate chronic methylmercury poisoning. Uchino reported that in 63 out of 77 certified Minamata disease patients (82%), changes in the range of sensory disturbance were observed [16]. The functions of the brain cortex have plasticity and can be affected by other parts of central nervous system, which can lead to fluctuations in signs and symptoms.

### 4.4. Late Onset of Methylmercury Poisoning

In this study, 65.0% of the subjects in the four exposed areas developed the first symptoms after 1968 (Table 12, Figure 7), when the dumping of polluted waste-water from the Chisso Cooperation stopped. After the appearance of the first symptom, the order of subsequent symptoms appeared gradually in the following order: muscle cramps, four-limb numbness, difficulty in fine finger tasks, stumbling tendency, and limited peripheral vision (Table 12). When we consider that most of the subjects had been exposed to methylmercury in or before 1968, there was a delay in the appearance of symptoms. In 1991, The Central Environmental Pollution Council of the Environment Agency (the predecessor of the present Ministry of Environment) (Japan) reported without evidence, “the period from methylmercury exposure to the onset is ordinarily supposed to be one month, at least within one year” [17]. Although this opinion is held by the Japanese administration even now, the reality is completely different. In the same year 1991, Igata, the chairman of the Council, wrote “roughly speaking, such late onset and late progression are limited to some patients and the peak was reached before 1975” [18], which was 7 years after the Chisso company stopped releasing contaminated wastewater in 1968 and also a time when cases with an acute onset of Minamata disease were rarely found. The true latency period is much longer.

Even in acute cases of maximum methylmercury exposure in Iraq, the mean latent periods ranged from 16 to 38 days [19]. In the experiments with monkeys, a latency period of 6 years was reported [20]. Evans et al. conducted a long-term study on nonhuman primates and demonstrated that the latency period was dose dependent [21,22]. Our data support the thesis that the latency period became longer as the exposure became milder (Table 13, Figure 9). The longest latency from exposure to symptomatic onset has not been determined. According to our data, the first symptom may already have been present before 2008, one year before the survey (Figure 7 and Figure 8), which explains that it is difficult to determine the longest latency period.

### 4.5. How Far Had the Methylmercury Pollution Spread?

Many of the subjects in the Northern Area (41.6%) had not lived in the designated area, but the pattern of signs and symptoms were almost the same as the Minamata Area (4.6%), Southern Area (7.7%), and Other Areas (5.3%) (Table 2). The scores of signs and symptoms in the non-designated area were almost the same as the designated area (Table 10). These data show that residents in the non-designated area had similar health effects from methylmercury and the spread of Minamata disease was larger than previously thought. At least, within the area where fish and shellfish can be obtained daily, adverse health effects caused by methylmercury may have occurred.

### 4.6. What Is the Longest Time, After Methylmercury Exposure, That Late Developing Symptoms Can Appear?

Similar patterns of signs and symptoms were observed in BA1968 (Table 15, Figure 11, Figure 12 and Figure 13) as same as in the elderly subjects. The scores of signs and symptoms in BA1968 were also similar to those of subjects in the designated area, and greater than the Control Area (Table 16). Figure 14 shows the development of methylmercury-related symptoms. The data indicates that the detrimental effects on health from methylmercury poisoning had continued to spread, even after the release of polluted waste-water had been stopped in 1968.

The Central Environmental Pollution Council of the Environment Agency, without any evidence, stated in 1991, “Since 1969, the possibilities of being exposed to levels of methylmercury that can cause Minamata disease no longer exist”. Our study shows that this statement in 1991 was incorrect.

It is difficult to determine whether subjects of BA1968 (as displayed in Figure 14) had developed their symptoms due to continued exposure after 1968 or if they were late developing symptoms. What we can say is that it is difficult to determine at which time, after chronic or continuous methylmercury exposure, that a person, not showing health problems, can be judged to be safe from late developing Minamata Disease symptoms.

### 4.7. Signs and Symptoms in Subjects Whose Sensory Disturbance Was Not Recognized

In Japan, there are a lot of people who have been exposed methylmercury and who have some neurological abnormalities. Four-limb sensory disturbance has been supposed to be the minimum neurological abnormality in Japan [1]. Outside of Japan, epidemiological studies have not seen such extreme neurological signs, but other more mild or latent neurocognitive and behavioral symptoms [23,24].

This study showed that subjects without sensory disturbance had experienced many other subjective symptoms and objective abnormalities similar to those of subjects with sensory disturbance (Table 17, Figure 15, Figure 16 and Figure 17). This means that there is a range of varieties of symptoms from methylmercury poisoning. Although sensory disturbance is important in methylmercury poisoning, the recognition of health problems caused by methylmercury should not be limited to cases with sensory disturbance.

### 4.8. Other Problems

There are some limitations in this study. Firstly, this study was a cross-sectional study and participants in this study were limited to subjects who had volunteered to be tested. Therefore, it is obvious that our data may not concur with the data of the general population in these areas. But our data does show the spread of methylmercury poisoning along Shiranui Seashore.

Secondly, the lifestyle and occupations were not the same among the four groups. But the high prevalence of the specific complaints and neurological findings of Minamata disease and the similar patterns of such prevalence of symptoms and neurological signs support our opinion.

## 5. Conclusions

The effects of methylmercury poisoning on human health had spread outside of the central area and could have still been caused until recently. By using the frequency of fish ingestion as an indirect indication of methylmercury exposure, a dose-response relationship was confirmed for methylmercury pollution in Minamata. The latency period from exposure of mercury to the onset of symptoms was much longer than previously thought, and the latency period increased as the exposure levels decreased. Further investigations on the health effects of methylmercury over a wider area, covering longer periods, and on a wider range of signs and symptoms must continue in the future.

## Figures and Tables

**Table 1:** Surveyed area and designated area.

	Designated Area	Non-Designated Area
Control Area		around Fukuoka City
		around Kumamoto City
		around Kagoshima City
Minamata Area (Kumamoto Prefecture)	Minamata City	
	Tsunagi Town	
	Ashikita Town *	
Northern Area (Kumamoto Prefecture)	Goshonoura district, Amakusa City **	Other districts of Amakusa City **
	Ryuugatake district, Kami-Amakusa City ***	Other districts of Kami-Amakusa City ***
	Futamisuguchi district, Yatsushiro City	Other districts of Yatsushiro City
		Uki City
		Hikawa Town
Southern Area (Kagoshima Prefecture)	All ex-Izumi City district, Izumi City ****	Other districts of Izumi City ****
	Euchi, Ookubo, Kamizuru, Shimozuru, and Shibabiki of ex-Takaono Town district, Izumi City ****	
	Shimomyo district of ex-Noda Town, Izumi City ****	
	All ex-Azuma Town district, Nagashima Town *****	Other districts (All ex-Nagashima Town) of Nagashima Town *****
	Wakimoto and Akasegawa districts of Akune City	Other districts of Akune City
Other Areas		Other districts of Kumamoto Prefecture
		Other districts of Kagoshima Prefecture
		Other Prefectures

* On 1 January 2005, ex-Ashikita Town and ex-Tanoura Town merged and became Ashikita Town; ** On 27 March 2006, ex-Goshonoura Town, 2 cities, and 7 other towns merged and became Amakusa City; *** On 31 March 2004, ex-Ryuugatake Town and 3 other towns merged and became Kami-Amakusa City; **** On 13 March 2006, ex-Izumi City, ex-Noda Town, and ex-Takaono Town merged and became Izumi City; ***** On 20 March 2006, ex-Azuma Town and ex-Nagashima Town merged and became Nagashima Town.

**Table 2:** Demographic characteristics of subjects in each area (*n* = 1115).

	Control Area	Minamata Area	Northern Area	Southern Area	Other Areas
	(*n* = 142)	(*n* = 259)	(*n* = 279)	(*n* = 246)	(*n* = 189)
Sex, *n* (%)					
Male	56 (39.4)	136 (52.5)	147 (52.7)	121 (49.2)	88 (46.6)
Female	86 (60.6)	123 (47.5)	132 (47.3)	125 (50.8)	101 (53.4)
Age					
Mean ± SD	62.0 ± 10.5	62.5 ± 13.6	64.0 ± 11.1	63.0 ± 11.1	58.6 ± 9.9
Range (min–max)	36–86	33–92	33–90	36–88	34–82
Residential history in designated area (DA) more than 1 year, *n* (%)					
In DA > 1 year (DA)	3 (2.1)	234 (90.3)	156 (55.9)	222 (90.2)	174 (92.1)
Not in DA > 1 year (NDA)	139 (97.9)	12 (4.6)	116 (41.6)	19 (7.7)	10 (5.3)
Born after 1968 (BA1968)	0 (0.0)	13 (5.0)	7 (2.5)	5 (2.0)	5 (2.6)
Smoking, *n* (%)					
Non-smoker	109 (77.3)	198 (76.4)	226 (81.0)	185 (75.2)	133 (70.4)
Smoker	32 (22.7)	61 (23.6)	53 (19.0)	61 (24.8)	56 (29.6)
Alcohol drinking, *n* (%)					
Non-drinker	72 (51.1)	136 (52.5)	148 (53.0)	129 (52.4)	98 (51.9)
Drinker	69 (48.9)	123 (47.5)	131 (47.0)	117 (47.6)	91 (48.1)
Frequency of fish intake, *n* (%)					
Three times a day	6 (4.4)	95 (36.7)	166 (59.5)	99 (40.2)	99 (52.4)
Twice a day	7 (5.1)	61 (23.6)	70 (25.1)	94 (38.2)	46 (24.3)
Once a day	27 (19.9)	59 (22.8)	27 (9.7)	35 (14.2)	25 (13.2)
More than once a week	63 (46.3)	35 (13.5)	13 (4.7)	16 (6.5)	15 (7.9)
Less than once a week	33 (24.3)	9 (3.5)	3 (1.1)	2 (0.8)	4 (2.1)
Occupation, *n* (%)					
Fishermen (subject)	0 (0.0)	7 (2.7)	82 (29.4)	32 (13.0)	10 (5.3)
Fishermen (subject’s parent)	2 (1.4)	28 (10.8)	153 (54.8)	78 (31.7)	60 (31.7)
Complications, *n* (%)					
Hypertension	40 (28.2)	85 (32.8)	118 (42.3)	102 (41.5)	43 (22.8)
Renal diseases	3 (2.1)	17 (6.6)	10 (3.6)	13 (5.3)	15 (7.9)
Liver diseases	6 (4.2)	20 (7.7)	17 (6.1)	20 (8.1)	14 (7.4)
Respiratory diseases	12 (8.5)	14 (5.4)	8 (2.9)	6 (2.4)	13 (6.9)
Diabetes Mellitus	3 (2.1)	17 (6.6)	37 (13.3)	24 (9.8)	15 (7.9)
Orthopedic diseases	13 (9.2)	60 (23.2)	72 (25.8)	62 (25.2)	52 (27.5)
Malignant diseases	7 (4.9)	12 (4.6)	11 (3.9)	17 (6.9)	11 (5.8)
History of application for Minamata disease, *n* (%)	0 (0.0)	33 (12.7)	24 (8.6)	38 (15.4)	17 (9.0)
Family history of Minamata disease, *n* (%)	0 (0.0)	164 (63.3)	108 (38.7)	123 (50.0)	151 (79.9)
Have witnessed abnormal animal behavior, *n* (%)	No Data	100 (38.6)	103 (36.9)	108 (43.9)	76 (40.2)

**Table 3:** Prevalence of symptoms (Always) and adjusted * odds ratios (OR) for the association between area and symptoms (*n* = 1115).

	Control Area	Minamata Area	Northern Area	Southern Area	Other Areas
Sensory numbness in both hands					
case/N (%)	3/138 (2.2)	98/259 (37.8)	116/278 (41.7)	126/246 (51.2)	73/189 (38.6)
OR (95% CI)	1 (reference)	27 (8.3–87)	30 (9.4–98)	46 (14–148)	29 (9.0–96)
Sensory numbness in both legs					
case/N (%)	1/139 (0.7)	98/259 (37.8)	125/279 (44.8)	126/246 (51.2)	68/189 (36.0)
OR (95% CI)	1 (reference)	80 (11–586)	100 (14–731)	140 (19–1022)	86 (12–633)
Perioral numbness **					
case/N (%)	0/107 (0.0)	15/259 (5.8)	25/279 (9.0)	31/246 (12.6)	14/189 (7.4)
OR (95% CI)	1 (reference)	5.8 (0.7–45)	8.8 (1.2–66)	13.9 (1.9–104)	9.6 (1.2–75)
Headache **					
case/N (%)	0/137 (0.0)	44/259 (17.0)	52/279 (18.6)	59/246 (24)	47/189 (24.9)
OR (95% CI)	1 (reference)	31 (4.2–228)	35 (4.7–254)	47 (6.4–344)	49 (6.6–358)
Muscle cramps					
case/N (%)	5/137 (3.6)	62/259 (23.9)	65/279 (23.3)	59/245 (24.1)	42/189 (22.2)
OR (95% CI)	1 (reference)	8.5 (3.3–22)	8.1 (3.2–21)	8.5 (3.3–22)	8.3 (3.2–22)
Limited peripheral vision					
case/N (%)	1/138 (0.7)	67/259 (25.9)	91/279 (32.6)	94/246 (38.2)	59/72 (81.9)
OR (95% CI)	1 (reference)	47 (6.5–347)	63 (8.6–457)	84 (12–614)	71 (9.6–520)
Difficulty in hearing					
case/N (%)	12/137 (8.8)	90/259 (34.7)	106/278 (38.1)	130/246 (52.8)	71/189 (37.6)
OR (95% CI)	1 (reference)	5.1 (2.7–9.9)	5.5 (2.9–11)	11 (5.9–22)	7.2 (3.7–14)
Difficulty in smelling					
case/N (%)	1/139 (0.7)	45/259 (17.4)	58/279 (20.8)	46/245 (18.8)	31/189 (16.4)
OR (95% CI)	1 (reference)	29 (3.9–213)	36 (5.0–266)	32 (4.4–238)	31 (4.1–228)
Difficulty in tasting **					
case/N (%)	0/140 (0.0)	34/259 (13.1)	50/279 (17.9)	46/245 (18.8)	23/189 (12.2)
OR (95% CI)	1 (reference)	20 (2.7–146)	28 (3.9–208)	31 (4.2–226)	20 (2.7–151)
Stumbling tendency **					
case/N (%)	0/106 (0.0)	72/259 (27.8)	99/279 (35.5)	89/246 (36.2)	48/189 (25.4)
OR (95% CI)	1 (reference)	41 (5.6–304)	59 (8.0–429)	63 (8.6–463)	45 (6.1–336)
Staggering **					
case/N (%)	0/107 (0.0)	57/259 (22.0)	76/278 (27.3)	73/246 (29.7)	33/189 (17.5)
OR (95% CI)	1 (reference)	29 (3.9–212)	38 (5.2–280)	46 (6.2–337)	29 (3.8–215)
Difficulty in fine finger tasks **					
case/N (%)	0/140 (0.0)	94/259 (36.3)	136/279 (48.7)	139/246 (56.5)	87/189 (46.0)
OR (95% CI)	1 (reference)	77 (11–562)	128 (18–930)	182 (25–1326)	135 (18–987)
Difficulty in buttoning **					
case/N (%)	0/140 (0.0)	52/259 (20.1)	73/279 (26.2)	53/245 (21.6)	28/189 (14.8)
OR (95% CI)	1 (reference)	31 (4.2–228)	43 (5.9–319)	36 (4.9–267)	30 (4.0–228)
Dropping things held in the hand **					
case/N (%)	0/140 (0.0)	39/259 (15.1)	60/279 (21.5)	56/246 (22.8)	22/189 (11.6)
OR (95% CI)	1 (reference)	24 (3.2–176)	36 (4.9–266)	41 (5.6–303)	22 (3.0–170)
Difficulty in speaking words or sentences well **					
case/N (%)	0/140 (0.0)	29/259 (11.2)	41/279 (14.7)	41/246 (16.7)	16/189 (8.5)
OR (95% CI)	1 (reference)	17 (2.3–125)	23 (3.1–167)	28 (3.8–206)	15 (2.0–118)
Postural hand tremor					
case/N (%)	2/138 (1.4)	41/259 (15.8)	50/279 (17.9)	48/246 (19.5)	31/189 (16.4)
OR (95% CI)	1 (reference)	12 (2.9–51)	14 (3.2–57)	16 (3.8–67)	16 (3.7–68)
Swaying or dizziness **					
case/N (%)	0/140 (0.0)	10/259 (3.9)	22/279 (7.9)	15/246 (6.1)	14/189 (7.4)
OR (95% CI)	1 (reference)	5.5 (0.7–44)	12 (1.5–88)	9.1 (1.2–70)	13 (1.7–105)
General fatigue					
case/N (%)	1/140 (0.7)	99/259 (38.2)	112/279 (40.1)	131/246 (53.3)	74/189 (39.2)
OR (95% CI)	1 (reference)	90 (12–657)	98 (14–714)	166 (23–1205)	94 (13–686)
Lack of motivation to do things					
case/N (%)	1/140 (0.7)	43/259 (16.6)	55/279 (19.7)	63/246 (25.6)	40/189 (21.2)
OR (95% CI)	1 (reference)	32 (4.4–237)	40 (5.5–294)	56 (7.6–409)	46 (6.2–341)
Losing your train of thought during conversations **					
case/N (%)	0/139 (0.0)	32/259 (12.4)	29/279 (10.4)	44/246 (17.9)	17/189 (9.0)
OR (95% CI)	1 (reference)	20 (2.6–146)	16 (2.1–118)	31 (4.1–225)	15 (2.0–118)
Forgetfulness					
case/N (%)	1/140 (0.7)	71/259 (27.4)	89/279 (31.9)	93/246 (37.8)	47/189 (24.9)
OR (95% CI)	1 (reference)	54 (7.4–396)	65 (8.9–473)	89 (12–651)	57 (7.7–419)
Irritation **					
case/N (%)	0/140 (0.0)	63/259 (24.3)	87/279 (31.2)	74/246 (30.1)	50/189 (26.5)
OR (95% CI)	1 (reference)	46 (6.3–337)	65 (9.0–475)	61 (8.4–446)	51 (6.9–373)
Anxiety **					
case/N (%)	0/106 (0.0)	78/259 (30.1)	77/279 (27.6)	72/246 (29.3)	56/189 (29.6)
OR (95% CI)	1 (reference)	48 (6.6–354)	43 (5.8–312)	46 (6.2–335)	48 (6.5–351)

* Adjusted for age, sex, diabetes mellitus and orthopedic diseases; ** When prevalence of the control group was zero, we postulated that a positive finding was found in the eldest subject in the control group and calculated the OR and 95% confidence interval.

**Table 4:** Correlation of prevalence of symptoms (Always) among each area.

Correlation/(*p*-value)	Control Area	Minamata Area	Northern Area	Southern Area
Minamata Area	0.3641	1.0000		
(*p*-value)	(0.0876)			
Northern Area	0.2751	0.9611	1.0000	
(*p*-value)	(0.2040)	(0.0000)		
Southern Area	0.3866	0.9540	0.9581	1.0000
(*p*-value)	(0.0684)	(0.0000)	(0.0000)	
Other Areas	0.3609	0.9417	0.9246	0.9460
(*p*-value)	(0.0906)	(0.0000)	(0.0000)	(0.0000)

**Table 5:** Prevalence of symptoms (Always and Sometimes) and adjusted * odds ratios (OR) for the association between area and symptoms (*n* = 1115).

	Control Area	Minamata Area	Northern Area	Southern Area	Other Areas
Sensory numbness in both hands					
case/N (%)	10/138 (7.2)	221/259 (85.3)	244/278 (87.8)	223/246 (90.7)	160/189 (84.7)
OR (95% CI)	1 (reference)	79 (38–165)	97 (46–206)	130 (59–284)	75 (35–161)
Sensory numbness in both legs					
case/N (%)	10/139 (7.2)	219/259 (84.6)	237/279 (84.9)	215/246 (87.4)	159/189 (84.1)
OR (95% CI)	1 (reference)	77 (37–161)	75 (36–156)	94 (44–201)	81 (37–175)
Perioral numbness **					
case/N (%)	0/107 (0.0)	104/259 (40.2)	136/279 (48.7)	118/246 (48.0)	81/189 (42.9)
OR (95% CI)	1 (reference)	73 (10–533)	104 (14–760)	101 (14–738)	87 (12–636)
Headache					
case/N (%)	31/137 (22.6)	208/259 (80.3)	219/279 (78.5)	198/246 (80.5)	154/189 (81.5)
OR (95% CI)	1 (reference)	17 (10–29)	16 (9.8–28)	18 (10–30)	17 (9.7–30)
Muscle cramps					
case/N (%)	53/137 (38.7)	237/259 (91.5)	256/279 (91.8)	220/245 (89.8)	172/189 (91.0)
OR (95% CI)	1 (reference)	18 (10–32)	19 (11–33)	15 (8.4–25)	17 (9.2–32)
Limited peripheral vision					
case/N (%)	10/138 (7.2)	171/259 (66.0)	198/279 (71.0)	192/246 (78.0)	132/189 (69.8)
OR (95% CI)	1 (reference)	25 (12–49)	29 (15–59)	44 (22–90)	31 (15–63)
Difficulty in hearing					
case/N (%)	25/137 (18.2)	178/259 (68.7)	212/278 (76.3)	196/246 (79.7)	133/189 (70.4)
OR (95% CI)	1 (reference)	9.8 (5.8–16)	13 (8.0–23)	17 (10–30)	12 (6.8–21)
Difficulty in smelling					
case/N (%)	7/139 (0.5)	124/259 (47.9)	137/279 (49.1)	139/245 (56.7)	96/189 (50.8)
OR (95% CI)	1 (reference)	17 (7.8–39)	18 (8.1–40)	25 (11–55)	20 (8.8–45)
Difficulty in tasting					
case/N (%)	3/140 (2.1)	119/259 (45.9)	148/279 (53.0)	140/245 (57.1)	95/189 (50.3)
OR (95% CI)	1 (reference)	39 (12–126)	51 (16–166)	61 (19–196)	47 (14–152)
Stumbling tendency					
case/N (%)	20/106 (18.9)	213/259 (82.2)	250/279 (89.6)	217/246 (88.2)	165/189 (87.3)
OR (95% CI)	1 (reference)	25 (13–46)	42 (22–81)	37 (19–72)	39 (20–78)
Staggering					
case/N (%)	10/107 (9.3)	197/259 (76.1)	227/278 (81.7)	205/246 (83.3)	149/189 (78.8)
OR (95% CI)	1 (reference)	35 (17–73)	47 (22–98)	53 (25–113)	45 (21–97)
Difficulty in fine finger tasks					
case/N (%)	11/140 (7.9)	195/259 (75.3)	224/279 (80.3)	215/246 (87.4)	148/189 (78.3)
OR (95% CI)	1 (reference)	36 (18–71)	46 (23–92)	81 (39–167)	45 (22–93)
Difficulty in buttoning **					
case/N (%)	0/140 (0.0)	139/259 (53.7)	177/279 (63.4)	149/245 (60.8)	106/189 (56.1)
OR (95% CI)	1 (reference)	158 (22–1147)	224 (31–1629)	211 (29–1536)	199 (27–1458)
Dropping things held in the hand					
case/N (%)	9/140 (6.4)	173/259 (66.8)	212/279 (76.0)	187/246 (76.0)	139/189 (73.5)
OR (95% CI)	1 (reference)	33 (16–68)	51 (24–106)	50 (24–106)	47 (22–100)
Difficulty in speaking words or sentences well					
case/N (%)	4/140 (2.9)	136/259 (52.5)	157/279 (56.3)	154/246 (62.6)	101/189 (53.4)
OR (95% CI)	1 (reference)	39 (14–108)	46 (16–128)	60 (21–167)	43 (15–122)
Postural hand tremor					
case/N (%)	7/138 (5.1)	160/259 (61.8)	188/279 (67.4)	171/246 (69.5)	116/189 (61.4)
OR (95% CI)	1 (reference)	30 (13–66)	37 (17–83)	42 (19–94)	31 (14–70)
Swaying or dizziness					
case/N (%)	8/140 (5.7)	117/259 (45.2)	157/279 (56.3)	138/246 (56.1)	101/189 (53.4)
OR (95% CI)	1 (reference)	14 (6.6–30)	22 (10–47)	22 (10–47)	20 (9.2–43)
General fatigue					
case/N (%)	30/140 (21.4)	234/259 (90.3)	253/279 (90.7)	230/246 (93.5)	171/189 (90.5)
OR (95% CI)	1 (reference)	43 (23–79)	46 (25–85)	65 (33–128)	37 (19–71)
Lack of motivation to do things					
case/N (%)	31/140 (22.1)	202/259 (78.0)	233/279 (83.5)	215/246 (87.4)	153/189 (81.0)
OR (95% CI)	1 (reference)	15 (9.0–25)	22 (13–38)	30 (17–52)	16 (9.2–28)
Losing your train of thought during conversations					
case/N (%)	11/139 (7.9)	169/259 (65.3)	204/279 (73.1)	183/246 (74.4)	125/189 (66.1)
OR (95% CI)	1 (reference)	24 (12–46)	34 (17–67)	36 (18–72)	26 (13–51)
Forgetfulness					
case/N (%)	79/140 (56.4)	240/259 (92.7)	257/279 (92.1)	235/246 (95.5)	176/189 (93.1)
OR (95% CI)	1 (reference)	11 (6.2–20)	10 (5.4–17)	18 (8.8–37)	13 (6.4–25)
Irritation					
case/N (%)	46/140 (32.9)	204/259 (78.8)	242/279 (86.7)	214/246 (87.0)	164/189 (86.8)
OR (95% CI)	1 (reference)	8.0 (5.0–13)	14 (8.6–24)	14 (8.5–24)	13 (7.6–23)
Anxiety					
case/N (%)	8/106 (7.5)	205/259 (79.2)	225/279 (80.6)	209/246 (85.0)	155/189 (82.0)
OR (95% CI)	1 (reference)	49 (23–109)	55 (25–122)	74 (33–166)	60 (27–136)

* Adjusted for age, sex, diabetes mellitus and orthopedic diseases; ** When prevalence of the control group was zero, we postulated that a positive finding was found in the eldest subject in the control group and calculated the OR and 95% confidence interval.

**Table 6:** Correlation of prevalence of symptoms (Always and Sometimes) among each area.

Correlation/(*p*-Value)	Control Area	Minamata Area	Northern Area	Southern Area
Minamata Area	0.6921	1.0000		
(*p*-value)	(0.0003)			
Northern Area	0.6708	0.9778	1.0000	
(*p*-value)	(0.0005)	(0.0000)		
Southern Area	0.6501	0.9787	0.9786	1.0000
(*p*-value)	(0.0008)	(0.0000)	(0.0000)	
Other Areas	0.7046	0.9875	0.9849	0.9795
(*p*-value)	(0.0002)	(0.0000)	(0.0000)	(0.0000)

**Table 7:** Prevalence of neurological findings and adjusted* odds ratios (OR) for the association between area and neurological findings (*n* = 1115).

	Control Area	Minamata Area	Northern Area	Southern Area	Other Areas
Dysarthria					
case/N (%)	2/141 (1.4)	34/256 (13.3)	37/276 (13.4)	41/242 (16.9)	22/186 (11.8)
OR (95% CI)	1 (reference)	9.7 (2.3–41)	9.3 (2.2–39)	13 (3.1–55)	9.5 (2.2–41)
Hearing loss					
case/N (%)	10/141 (7.1)	59/258 (22.9)	54/279 (19.4)	65/244 (26.6)	27/186 (14.5)
OR (95% CI)	1 (reference)	3.5 (1.7–7.1)	2.6 (1.3–5.4)	4.4 (2.1–9.0)	2.6 (1.2–5.7)
Visual field disturbance **					
case/N (%)	0/142 (0.0)	51/255 (20.0)	66/276 (23.9)	61/243 (25.1)	49/187 (26.2)
OR (95% CI)	1 (reference)	33 (4.4–240)	39 (5.4–288)	44 (6.0–322)	53 (7.2–392)
Normal gait disturbance (distinct) **					
case/N (%)	0/137 (0.0)	24/238 (10.1)	25/255 (9.8)	36/221 (16.3)	15/174 (8.6)
OR (95% CI)	1 (reference)	12 (1.5–87)	11 (1.5–83)	22 (3.0–164)	14 (1.8–107)
Normal gait disturbance (mild-distinct) **					
case/N (%)	0/137 (0.0)	50/238 (21.0)	41/255 (16.1)	52/221 (23.5)	26/174 (14.9)
OR (95% CI)	1 (reference)	30 (4.0–222)	21 (2.8–156)	39 (5.3–292)	32 (4.2–242)
Tandem gait disturbance (distinct)					
case/N (%)	3/141 (2.1)	32/248 (12.9)	41/271 (15.1)	32/241 (13.3)	14/186 (7.5)
OR (95% CI)	1 (reference)	6.0 (1.8–21)	8.0 (2.4–27)	7.2 (2.1–24)	5.7 (1.6–21)
Tandem gait disturbance (mild-distinct)					
case/N (%)	15/141 (10.6)	115/248 (46.4)	151/271 (55.7)	135/241 (56.0)	84/186 (45.2)
OR (95% CI)	1 (reference)	8.0 (4.3–15)	11 (6.0–20)	12 (6.7–23)	10 (5.3–19)
Mann test (distinct)					
case/N (%)	3/109 (2.8)	67/252 (26.6)	76/268 (28.4)	62/242 (25.6)	39/187 (20.9)
OR (95% CI)	1 (reference)	12 (3.7–40)	13 (3.9–42)	12 (3.5–39)	11 (3.3–37)
Mann test (mild-distinct)					
case/N (%)	26/109 (23.9)	153/252 (60.7)	176/268 (65.7)	151/242 (62.4)	114/187 (61.0)
OR (95% CI)	1 (reference)	5.4 (3.2–9.2)	6.2 (3.6–11)	5.6 (3.3–9.5)	6.3 (3.6–11)
One-foot standing abnormality (eyes open) (distinct)					
case/N (%)	2/141 (1.4)	43/253 (17.0)	61/269 (22.7)	47/242 (19.4)	27/189 (14.3)
OR (95% CI)	1 (reference)	14 (3.3–60)	21 (5.0–89)	18 (4.3–78)	19 (4.4–85)
One-foot standing abnormality (eyes open) (mild-distinct)					
case/N (%)	16/141 (11.3)	121/253 (47.8)	151/269 (56.1)	125/242 (51.7)	85/189 (45.0)
OR (95% CI)	1 (reference)	8.4 (4.6–15)	11 (6.0–20)	9.7 (5.3–18)	9.7 (5.2–18)
One-foot standing abnormality (eyes shut) (distinct)					
case/N (%)	16/142 (11.3)	128/252 (50.8)	135/269 (50.2)	115/243 (47.3)	78/188 (41.5)
OR (95% CI)	1 (reference)	9.7 (5.3–18)	8.4 (4.6–15)	8.0 (4.4–15)	8.2 (4.4–15)
One-foot standing abnormality (eyes shut) (mild-distinct)					
case/N (%)	75/142 (52.8)	202/252 (80.2)	228/269 (84.8)	195/243 (80.2)	150/188 (79.8)
OR (95% CI)	1 (reference)	4.5 (2.7–7.4)	5.1 (3.1–8.5)	3.9 (2.4–6.4)	4.9 (2.9–8.3)
Finger-nose test (eyes open) (distinct) **					
case/N (%)	0/140 (0.0)	10/254 (3.9)	13/264 (4.9)	13/233 (5.6)	11/183 (6.0)
OR (95% CI)	1 (reference)	5.2 (0.6–41)	6.5 (0.8–51)	7.8 (0.99995–61)	9.4 (1.2–75)
Finger-nose test (eyes open) (mild-distinct) **					
case/N (%)	0/140 (0.0)	32/254 (12.6)	61/264 (23.1)	50/233 (21.5)	29/183 (15.8)
OR (95% CI)	1 (reference)	19 (2.6–142)	39 (5.4–288)	37 (5.0–272)	29 (3.8–214)
Finger-nose test (eyes shut) (distinct) **					
case/N (%)	0/139 (0.0)	34/251 (13.5)	31/262 (11.8)	35/233 (15)	28/182 (15.4)
OR (95% CI)	1 (reference)	21 (2.9–157)	18 (2.5–137)	25 (3.4–185)	29 (3.8–215)
Finger-nose test (eyes shut) (mild-distinct)					
case/N (%)	3/139 (2.2)	69/251 (27.5)	95/262 (36.3)	86/233 (36.9)	52/182 (28.6)
OR (95% CI)	1 (reference)	17 (5.1–54)	24 (7.4–78)	26 (7.9–84)	20 (5.9–64)
Adiadokokinesis (distinct) **					
case/N (%)	0/135 (0.0)	11/245 (4.5)	17/270 (6.3)	19/228 (8.3)	15/178 (8.4)
OR (95% CI)	1 (reference)	5.3 (0.7–42)	7.4 (0.96–56)	10.3 (1.4–79)	11.8 (1.5–91)
Adiadokokinesis (mild-distinct)					
case/N (%)	3/135 (2.2)	40/245 (16.3)	85/270 (31.5)	61/228 (26.8)	43/178 (24.2)
OR (95% CI)	1 (reference)	7.9 (2.4–26)	18 (5.6–59)	15 (4.6–49)	16 (4.7–52)
Heel-knee test (distinct) **					
case/N (%)	0/135 (0.0)	17/226 (7.5)	15/239 (6.3)	19/207 (9.2)	9/163 (5.5)
OR (95% CI)	1 (reference)	9.8 (1.3–75)	7.8 (1.0–60)	12 (1.6–93)	8.4 (1.0–68)
Heel-knee test (mild-distinct)					
case/N (%)	3/135 (2.2)	60/226 (26.5)	66/239 (27.6)	68/207 (32.9)	49/163 (30.1)
OR (95% CI)	1 (reference)	16 (4.8–51)	16 (4.9–52)	21 (6.5–70)	22 (6.6–73)
Postural Hand tremor					
case/N (%)	6/136 (4.4)	48/259 (18.5)	58/279 (20.8)	56/246 (22.8)	38/189 (20.1)
OR (95% CI)	1 (reference)	4.7 (2.0–11)	5.3 (2.2–13)	6.1 (2.5–15)	5.6 (2.3–14)
Touch disturbance (four-limb peripheral)					
case/N (%)	1/142 (0.7)	159/259 (61.4)	204/279 (73.1)	164/246 (66.7)	126/189 (66.7)
OR (95% CI)	1 (reference)	218 (30–1581)	368 (50–2678)	271 (37–1977)	265 (36–1944)
Touch disturbance (perioral) **					
case/N (%)	0/142 (0.0)	63/259 (24.3)	71/279 (25.4)	50/246 (20.3)	45/189 (23.8)
OR (95% CI)	1 (reference)	45 (6.2–331)	48 (6.6–353)	36 (4.9–264)	45 (6.1–330)
Touch disturbance (systemic) **					
case/N (%)	0/142 (0.0)	43/259 (16.6)	49/279 (17.6)	43/246 (17.5)	25/189 (13.2)
OR (95% CI)	1 (reference)	28 (3.9–209)	30 (4.1–223)	31 (4.2–225)	24 (3.2–179)
Pain disturbance (four-limb peripheral)					
case/N (%)	2/142 (1.4)	185/259 (71.4)	224/279 (80.3)	167/246 (67.9)	136/189 (72.0)
OR (95% CI)	1 (reference)	183 (44–760)	296 (71–1238)	153 (37–636)	183 (44–767)
Pain disturbance (perioral) **					
case/N (%)	0/142 (0.0)	103/259 (39.8)	104/279 (37.3)	65/246 (26.4)	60/189 (31.7)
OR (95% CI)	1 (reference)	93 (13–673)	83 (11–603)	50 (6.9–367)	67 (9.1–491)
Pain disturbance (systemic) **					
case/N (%)	0/142 (0.0)	56/259 (21.6)	73/279 (26.2)	56/246 (22.8)	38/189 (20.1)
OR (95% CI)	1 (reference)	38 (5.2–281)	49 (6.7–355)	41 (5.6–300)	37 (5.0–273)

* Adjusted for age, sex, diabetes mellitus and orthopedic diseases; ** When prevalence of the control group was zero, we postulated that a positive finding was found in the eldest subject in the control group and calculated the OR and 95% confidence interval.

**Table 8:** Correlation of prevalence of neurological findings among each area.

Correlation/(*p*-Value)	Control Area	Minamata Area	Northern Area	Southern Area
Minamata Area	0.6709	1.0000		
(*p*-value)	(0.0001)			
Northern Area	0.6327	0.9792	1.0000	
(*p*-value)	(0.0003)	(0.0000)		
Southern Area	0.6802	0.9716	0.9823	1.0000
(*p*-value)	(0.0001)	(0.0000)	(0.0000)	
Other Areas	0.6518	0.9778	0.9864	0.9771
(*p*-value)	(0.0002)	(0.0000)	(0.0000)	(0.0000)

**Table 9:** Score of signs and symptoms in each area (*n* = 1115).

	Control Area	Minamata Area	Northern Area	Southern Area	Other Areas
	(*n* = 142)	(*n* = 259)	(*n* = 279)	(*n* = 246)	(*n* = 189)
Symptom score (always)					
Mean ± SD	0.2 ± 0.5	5.1 ± 4.9	6.1 ± 5.0	6.9 ± 5.4	5.3 ± 4.9
Range (min–max)	0–3	0–22	0–23	0–22	0–23
Symptom score (always and sometimes)					
Mean ± SD	3.2 ± 3.0	21.2 ± 9.2	23.3 ± 9.0	24.7 ± 9.2	21.9 ± 9.4
Range (min–max)	0–15	2–45	1–46	3–45	2–46
Cranial nerve score					
Mean ± SD	0.2 ± 0.6	1.1 ± 1.7	1.1 ± 1.5	1.4 ± 1.8	1 ± 1.5
Range (min–max)	0–2	0–6	0–6	0–6	0–6
Upper, lower ataxia and tremor score					
Mean ± SD	0.1 ± 0.4	1.0 ± 1.2	1.3 ± 1.5	1.3 ± 1.5	1.1 ± 1.4
Range (min–max)	0–3	0–5	0–5	0–5	0–5
Truncal ataxia score					
Mean ± SD	0.9 ± 1.0	2.5 ± 1.7	2.7 ± 1.6	2.7 ± 1.6	2.4 ± 1.6
Range (min–max)	0–3	0–5	0–5	0–5	0–5
Sensory score					
Mean ± SD	0.0 ± 0.2	2.4 ± 1.5	2.6 ± 1.5	2.2 ± 1.3	2.3 ± 1.2
Range (min–max)	0–2	0–6	0–6	0–6	0–5
Total neurological score					
Mean ± SD	1.2 ± 1.4	6.9 ± 4.5	7.7 ± 4.2	7.6 ± 4.3	6.8 ± 4.0
Range (min–max)	0–8	0–20	0–21	0–21	0–19

**Table 10:** Score of signs and symptoms in DA, NDA, and BA1968 (*n* = 1115).

	Control Area	DA	NDA	BA1968
Age (Mean ± SD)	62.0 ± 10.5	63.0 ± 11.3	63.6 ± 10.0	37.4 ± 2.3
(*n*)	(142)	(786)	(158)	(30)
Symptom score (always)				
Mean ± SD	0.2 ± 0.5	5.9 ± 5.2	6.1 ± 4.9	4.0 ± 5.0
Range (min–max)	0–3	0–23	0–23	0–19
Symptom score (always and sometimes)				
Mean ± SD	3.2 ± 3.0	22.9 ± 9.2	23.1 ± 9.2	19.7 ± 9.9
Range (min–max)	0–15	2–46	1–46	2–42
Cranial nerve score				
Mean ± SD	0.2 ± 0.6	1.2 ± 1.7	1.1 ± 1.6	0.4 ± 0.8
Range (min–max)	0–2	0–6	0–6	0–2
Upper, lower ataxia and tremor score				
Mean ± SD	0.1 ± 0.4	1.2 ± 1.4	1.3 ± 1.5	1.0 ± 1.2
Range (min–max)	0–3	0–5	0–5	0–4
Truncal ataxia score				
Mean ± SD	0.9 ± 1.0	2.6 ± 1.6	2.7 ± 1.7	1.7 ± 1.3
Range (min–max)	0–3	0–5	0–5	0–4
Sensory score				
Mean ± SD	0.0 ± 0.2	2.4 ± 1.4	2.4 ± 1.4	2.0 ± 1.7
Range (min–max)	0–2	0–6	0–6	0–6
Total neurological score				
Mean ± SD	1.2 ± 1.4	7.3 ± 4.3	7.5 ± 4.2	5.1 ± 3.8
Range (min–max)	0–8	0–21	0–20	0–13

**Table 11:** Frequency of fish ingestion and scores (*n* = 973).

Frequency	*n*		Age	Symptom (Always) Score	Symptom (Always & Sometimes) Score	Total Neurological Score
3/day	459	Mean ± SD	62.9 ± 11.6	6.7 ± 5.5	24.4 ± 9.3	7.9 ± 4.3
		Min–Max	33–90	0–23	3–46	0–21
2/day	271	Mean ± SD	62.2 ± 11.5	5.9 ± 4.7	23.0 ± 8.8	7.1 ± 4.3
		Min–Max	36–92	0–21	1–44	0–21
1/day	146	Mean ± SD	61.9 ± 12.7	4.8 ± 4.7	20.8 ± 8.7	7.0 ± 4.3
		Min–Max	34–89	0–21	2–44	0–20
≥1/week	79	Mean ± SD	61.0 ± 11.3	3.4 ± 3.7	17.0 ± 8.4	5.7 ± 3.7
		Min–Max	35–86	0–15	2–36	0–16
<1/week	18	Mean ± SD	58.9 ± 12.3	5.1 ± 4.6	20.7 ± 10	4.4 ± 3.3
		Min–Max	39–81	0–16	4–38	1–11
<1/day	97	Mean ± SD	60.6 ± 11.4	3.7 ± 3.9	17.7 ± 8.8	5.5 ± 3.6
(≥1/week & <1/week)		Min–Max	35–86	0–16	2–38	0–16
Total	973	Mean ± SD	62.3 ± 11.7	5.9 ± 5.1	22.8 ± 9.3	7.3 ± 4.3
		Min–Max	33–92	0–23	1–46	0–21

**Table 12:** Average onset year of symptoms and interval between the first symptom and the onset of each following symptom in each area.

	Minamata Area	Northern Area	Southern Area	Other Areas	Total of Polluted Areas
First symptom	1979.0 ± 14.8	1974.9 ± 16.1	1973.6 ± 14.2	1974.6 ± 13.7	1975.6 ± 15
case/N (%)	256/259 (98.8)	275/279 (98.6)	245/246 (99.6)	189/189 (100)	965/973 (99.2)
>1968/case (%)	185/256 (72.3)	175/275 (63.6)	143/245 (58.4)	124/189 (65.6)	627/965 (65.0)
Muscle cramps	1982.2 ± 15.1	1979.5 ± 15.6	1978.1 ± 15.1	1978.4 ± 14.1	1979.6 ± 15.1
case/N (%)	235/259 (90.7)	255/279 (91.4)	227/246 (92.3)	182/189 (96.3)	899/973 (92.4)
>1968/case (%)	185/235 (78.7)	192/255 (75.3)	157/227 (69.2)	139/182 (76.4)	673/899 (74.9)
First symptom-Muscle cramps	4.4 ± 8.8	5.5 ± 10.4	4.9 ± 8.6	4.2 ± 7.2	4.8 ± 8.9
Four limb numbness	1987.8 ± 14.2	1982.2 ± 15.9	1982.3 ± 14.5	1983.9 ± 13.9	1984 ± 14.9
case/N (%)	237/259 (91.5)	254/279 (91.0)	235/246 (95.5)	172/189 (91.0)	898/973 (92.3)
>1968/case (%)	205/237 (86.5)	205/254 (80.7)	189/235 (80.4)	151/172 (87.8)	750/898 (83.5)
First symptom-Four limb numbness	9.1 ± 12.5	8.1 ± 12.0	8.5 ± 11.2	9.4 ± 11.4	8.7 ± 11.8
Stumbling tendency	1990.7 ± 13.1	1989.2 ± 13.7	1987.9 ± 14.3	1986.8 ± 13.4	1988.8 ± 13.7
case/N (%)	188/259 (72.6)	216/279 (77.4)	196/246 (79.7)	144/189 (76.2)	744/973 (76.5)
>1968/case (%)	176/188 (93.6)	196/216 (90.7)	175/196 (89.3)	133/144 (92.4)	680/744 (91.4)
First symptom-Stumbling tendency	12.7 ± 13.2	15.4 ± 13.2	14.9 ± 14.0	14.1 ± 13.7	14.3 ± 13.5
Difficulty in fine finger tasks	1989.5 ± 13.6	1986.9 ± 14.4	1985.8 ± 13.5	1983.1 ± 13.8	1986.5 ± 14.0
case/N (%)	144/259 (55.6)	182/279 (65.2)	178/246 (72.4)	114/189 (60.3)	618/973 (63.5)
>1968/case (%)	135/144 (93.8)	161/182 (88.5)	157/178 (88.2)	95/114 (83.3)	548/618 (88.7)
First symptom-Difficulty in fine finger tasks	12.3 ± 12.7	14.4 ± 13.1	13.4 ± 12.9	12.1 ± 12.0	13.2 ± 12.7
Limited peripheral vision	1992.2 ± 13.8	1988.8 ± 14.6	1991.9 ± 10.9	1988.9 ± 12.7	1990.5 ± 13.2
case/N (%)	134/259 (51.7)	179/279 (64.2)	168/246 (68.3)	110/189 (58.2)	591/973 (60.7)
>1968/case (%)	126/134 (94.0)	160/179 (89.4)	165/168 (98.2)	103/110 (93.6)	554/591 (93.7)
First symptom-Limited peripheral vision	16.5 ± 13.6	16.9 ± 12.9	18.7 ± 13.1	16.8 ± 13.7	17.3 ± 13.3

**Table 13:** Average onset year of symptoms and interval from the first symptom in each category of fish ingestion (*n* = 965).

	3/day	2/day	1/day	≥1/week	<1/week
	(*n* = 455)	(*n* = 269)	(*n* = 144)	(*n* = 79)	(*n* = 18)
First symptom	1974.2 ± 15.1	1975.6 ± 14.6	1978.2 ± 14.8	1977.7 ± 15	1982.5 ± 15.2
>1968/case (%)	277/455 (60.9)	174/269 (64.7)	105/144 (72.9)	56/79 (70.9)	15/18 (83.3)
Muscle cramps	1978 ± 15.1	1980.6 ± 14.9	1981.7 ± 14.5	1980.9 ± 15.5	1986.4 ± 17
case/N (=965) (%)	428/455 (94.1)	255/269 (94.8)	133/144 (92.4)	69/79 (87.3)	14/18 (77.8)
>1968/case (%)	308/428 (72.0)	194/255 (76.1)	108/133 (81.2)	52/69 (75.4)	11/14 (78.6)
First symptom-Muscle cramps	4.6 ± 8.2	5.4 ± 10.4	4.9 ± 9.3	3.9 ± 6.3	5.7 ± 10.5
Four limb numbness	1981.3 ± 15.5	1984.6 ± 13.8	1987.5 ± 14.6	1990.2 ± 12.6	1991.8 ± 11.7
case/N (=965) (%)	430/455 (94.5)	251/269 (93.3)	129/144 (89.6)	73/79 (92.4)	15/18 (83.3)
>1968/case (%)	339/430 (78.8)	216/251 (86.1)	111/129 (86.0)	69/73 (94.5)	15/15 (100)
First symptom-Four limb numbness	7.6 ± 11.1	8.9 ± 11.6	9.6 ± 12.4	13.1 ± 14.4	9.5 ± 13.1
Stumbling tendency	1987.2 ± 13.9	1988.9 ± 14.1	1990.6 ± 12.5	1994.5 ± 11.5	1993.8 ± 10.5
case/N (=965) (%)	368/455 (80.9)	206/269 (76.6)	112/144 (77.8)	48/79 (60.8)	10/18 (55.6)
>1968/case (%)	332/368 (90.2)	185/206 (89.8)	105/112 (93.8)	48/48 (100)	10/10 (100)
First symptom-Stumbling tendency	14.3 ± 13.0	14.2 ± 13.8	13 ± 13.2	18.9 ± 16.2	14.5 ± 15.2
Difficulty in fine finger tasks	1985.4 ± 13.6	1986.3 ± 14.4	1989.1 ± 14.2	1989.1 ± 14.4	1996.9 ± 8.4
case/N (=965) (%)	320/455 (70.3)	168/269 (62.5)	82/144 (56.9)	40/79 (50.6)	8/18 (44.4)
>1968/case (%)	284/320 (88.8)	147/168 (87.5)	73/82 (89.0)	36/40 (90.0)	8/8 (100)
First symptom-Difficulty in fine finger tasks	13.1 ± 12.3	13 ± 12.7	13.9 ± 13.9	13.1 ± 13.7	12.5 ± 16.7
Limited peripheral vision	1989.7 ± 12.8	1990.8 ± 13.3	1991.8 ± 12.9	1991.3 ± 16.1	1993.8 ± 13.8
case/N (=965) (%)	302/455 (66.4)	165/269 (61.3)	80/144 (55.6)	36/79 (45.6)	8/18 (44.4)
>1968/case (%)	281/302 (93.0)	155/165 (93.9)	77/80 (96.3)	33/36 (91.7)	8/8 (100)
First symptom-Limited peripheral vision	17.5 ± 12.9	17.3 ± 13.7	16.3 ± 13.4	19.1 ± 14.3	13.1 ± 16.3

**Table 14:** Score of signs and onset of symptoms in each area.

	First Symptom	Muscle Cramps	Four Limb Numbness	Stumbling Tendency	Difficulty in Fine Finger Tasks	Limited Peripheral Vision
Symptom score (always)						
*R*^2^	0.0496	0.0329	0.0789	0.0453	0.0383	0.0567
*p*-value	(0.000)	(0.000)	(0.000)	(0.000)	(0.000)	(0.000)
Symptom score (always and sometimes)						
*R*^2^	0.0845	0.0612	0.1134	0.0641	0.0443	0.0716
*p*-value	(0.000)	(0.000)	(0.000)	(0.000)	(0.000)	(0.000)
Cranial nerve score						
*R*^2^	0.0193	0.0127	0.0222	0.0098	0.0105	0.0062
*p*-value	(0.000)	(0.000)	(0.000)	(0.004)	(0.006)	(0.031)
Upper, lower ataxia and tremor score						
*R*^2^	0.0298	0.0173	0.0438	0.0237	0.0121	0.0141
*p*-value	(0.000)	(0.000)	(0.000)	(0.000)	(0.004)	(0.002)
Truncal ataxia score						
*R*^2^	0.0143	0.0046	0.0291	0.0165	0.0115	0.008
*p*-value	(0.023)	(0.000)	(0.000)	(0.004)	(0.017)	(0.000)
Sensory score						
*R*^2^	0.0219	0.0163	0.0309	0.0272	0.022	0.0058
*p*-value	(0.000)	(0.000)	(0.000)	(0.000)	(0.000)	(0.035)
Total neurological score						
*R*^2^	0.0428	0.0248	0.0633	0.0385	0.0289	0.0189
*p*-value	(0.000)	(0.000)	(0.000)	(0.000)	(0.000)	(0.000)

**Table 15:** Demographic characteristics of subjects in younger subjects (*n* = 202).

	Control Area	BA1968	Designated Area	Total
	(*n* = 88)	(*n* = 30)	(*n* = 84)	(*n* = 202)
Sex, *n* (%)				
Male	40 (45.5)	21 (70.0)	44 (52.4)	105 (52.0)
Female	48 (54.6)	9 (30.0)	40 (47.6)	97 (48.0)
Age				
Mean ± SD	37.5 ± 6.0	37.4 ± 2.3	44.8 ± 2.3	40.5 ± 5.6
Range (min–max)	30–48	33–40	40–48	30–48
Smoking, *n* (%)				
Non-smoker	56 (65.1)	19 (63.3)	49 (58.3)	124 (62.0)
Smoker	30 (34.9)	11 (36.7)	35 (41.7)	76 (38.0)
Alcohol drinking, *n* (%)				
Non-drinker	41 (47.7)	11 (36.7)	30 (35.7)	82 (41.0)
Drinker	45 (52.3)	19 (63.3)	54 (64.3)	118 (59.0)
Frequency of fish intake, *n* (%)				
Three times a day	3 (3.4)	15 (50.0)	30 (35.7)	48 (23.8)
Twice a day	1 (1.1)	6 (20.0)	24 (28.6)	31 (15.4)
Once a day	9 (10.2)	5 (16.7)	21 (25.0)	35 (17.3)
More than once a week	53 (60.2)	2 (6.7)	8 (9.5)	63 (31.2)
Less than once a week	20 (22.7)	2 (6.7)	1 (1.2)	23 (11.4)
Occupation, *n* (%)				
Fishermen (subject)	0 (0.0)	1 (3.3)	5 (6.0)	6 (3.0)
Fishermen (subject’s parent)	1 (1.2)	9 (30.0)	23 (27.4)	33 (16.6)
Complications, *n* (%)				
Hypertension	1 (1.1)	0 (0.0)	8 (9.5)	9 (4.5)
Renal diseases	0 (0.0)	0 (0.0)	3 (3.6)	3 (1.5)
Liver diseases	1 (1.1)	0 (0.0)	2 (2.4)	3 (1.5)
Respiratory diseases	8 (9.1)	1 (3.3)	6 (7.1)	15 (7.4)
Diabetes Mellitus	0 (0.0)	1 (3.3)	2 (2.4)	3 (1.5)
Orthopedic diseases	3 (3.4)	3 (10)	14 (16.7)	20 (9.9)
Malignant diseases	0 (0.0)	0 (0.0)	1 (1.2)	1 (0.5)
History of application for inamata disease, *n* (%)	0 (0.0)	1 (3.3)	13 (15.5)	14 (6.9)
Family history of Minamata disease, *n* (%)	0 (0.0)	29 (96.7)	76 (90.5)	105 (52.0)
Have witnessed abnormal animal behavior, *n* (%)	No Data	9 (30.0)	27 (32.1)	36 (31.6)

**Table 16:** Score of signs and symptoms in younger subjects.

	Control Area	BA1968	Designated Area
Age (Mean ± SD)	37.5 ± 10.0	37.4 ± 10.0	44.8 ± 10.0
(*n*)	(88)	(30)	(84)
Symptom score (always)			
Mean ± SD	0.1 ± 0.4	4.0 ± 5.0	4.2 ± 4.3
Range (min–max)	0–3	0–19	0–19
Symptom score (always and sometimes)			
Mean ± SD	2.5 ± 2.9	19.7 ± 9.9	19.8 ± 9.1
Range (min–max)	0–16	2–42	2–42
Cranial nerve score			
Mean ± SD	0.0 ± 0.2	0.4 ± 0.8	0.5 ± 1.1
Range (min–max)	0–2	0–2	0–4
Upper, lower ataxia and tremor score			
Mean ± SD	0.0 ± 0.0	1.0 ± 1.2	0.6 ± 1.0
Range (min–max)	0–0	0–4	0–4
Truncal ataxia score			
Mean ± SD	0.2 ± 0.5	1.7 ± 1.3	1.7 ± 1.5
Range (min–max)	0–3	0–4	0–5
Sensory score			
Mean ± SD	0.0 ± 0.1	2.0 ± 1.7	2.2 ± 1.3
Range (min–max)	0–1	0–6	0–5
Total neurological score			
Mean ± SD	0.2 ± 0.7	5.1 ± 3.8	5.0 ± 3.3
Range (min–max)	0–5	0–13	0–16

**Table 17:** Demographic characteristics of subjects in each group (*n* = 1115).

	Control Area	Sensory Disturbance (−)	Sensory Disturbance (+)
	(*n* = 142)	(*n* = 91)	(*n* = 882)
Sex, *n* (%)			
Male	56 (39.4)	64 (70.3)	428 (48.5)
Female	86 (60.6)	27 (29.7)	454 (51.5)
Age			
Mean ± SD	62.0 ± 10.5	59.7 ± 13.3	62.6 ± 11.6
Range (min–max)	36–86	33–87	33–92
Residential history in designated area (DA) more than 1 year, *n* (%)			
In DA > 1 year (DA)	3 (2.1)	69 (75.8)	717 (81.3)
Not in DA > 1 year (NDA)	139 (97.9)	13 (14.3)	144 (16.3)
Born after 1968 (BA1968)	0 (0.0)	9 (5.0)	21 (2.4)
Smoking, *n* (%)			
Non-smoker	109 (77.3)	66 (72.5)	676 (81.0)
Smoker	32 (22.7)	25 (27.5)	206 (19.0)
Alcohol drinking, *n* (%)			
Non-drinker	72 (51.1)	43 (47.3)	468 (53.1)
Drinker	69 (48.9)	48 (52.7)	414 (47.0)
Frequency of fish intake, *n* (%)			
Three times a day	6 (4.4)	34 (37.4)	425 (48.2)
Twice a day	7 (5.1)	30 (33.0)	241 (27.3)
Once a day	27 (19.9)	12 (13.2)	134 (15.2)
More than once a week	63 (46.3)	13 (14.3)	66 (7.5)
Less than once a week	33 (24.3)	2 (2.2)	16 (1.8)
Occupation, *n* (%)			
Fishermen (subject)	0 (0.0)	13 (14.3)	118 (13.4)
Fishermen (subject’s parent)	2 (1.6)	28 (30.8)	291 (33.0)
Complications, *n* (%)			
Hypertension	40 (28.2)	28 (30.8)	320 (36.3)
Renal diseases	3 (2.1)	7 (7.7)	48 (5.4)
Liver diseases	6 (4.2)	10 (11.0)	61 (6.9)
Respiratory diseases	12 (8.5)	4 (4.4)	37 (4.2)
Diabetes Mellitus	3 (2.1)	9 (9.9)	84 (9.5)
Orthopedic diseases	13 (9.2)	22 (24.2)	224 (25.4)
Malignant diseases	7 (4.9)	3 (3.3)	48 (5.4)
History of application for Minamata disease, *n* (%)	0 (0.0)	3 (3.3)	109 (12.4)
Family history of Minamata disease, *n* (%)	0 (0.0)	48 (52.7)	498 (56.5)
Have witnessed abnormal animal behavior, *n* (%)	No Data	27 (29.7)	360 (40.8)

**Table 18:** Score of signs and symptoms in subjects with and without sensory disturbance (*n* = 1115).

	Control Area	Sensory Disturbance (−)	Sensory Disturbance (+)
Age (Mean ± SD)	62.0 ± 10.5	59.7 ± 13.3	62.6 ± 11.6
(*n*)	(142)	(91)	(882)
Symptom score (always)			
Mean ± SD	0.2 ± 0.5	4.0 ± 4.0	6.1 ± 5.2
Range (min–max)	0–3	0–16	0–23
Symptom score (always and sometimes)			
Mean ± SD	3.2 ± 3.0	18.0 ± 8.7	23.3 ± 9.2
Range (min–max)	0–15	2–38	1–46
Cranial nerve score			
Mean ± SD	0.2 ± 0.6	0.9 ± 1.4	1.2 ± 1.7
Range (min–max)	0–2	0–6	0–6
Upper, lower ataxia and tremor score			
Mean ± SD	0.1 ± 0.4	0.6 ± 1.0	1.2 ± 1.4
Range (min–max)	0–3	0–4	0–5
Truncal ataxia score			
Mean ± SD	0.9 ± 1.0	1.8 ± 1.7	2.7 ± 1.6
Range (min–max)	0–3	0–5	0–5
Sensory score			
Mean ± SD	0.0 ± 0.2	0.0 ± 0.0	2.6 ± 1.2
Range (min–max)	0–2	0–0	1–6
Total neurological score			
Mean ± SD	1.2 ± 1.4	3.4 ± 3.0	7.7 ± 4.2
Range (min–max)	0–8	0–15	1–21
