# Correction: Takaoka, S., et al. Survey of the Extent of the Persisting Effects of Methylmercury Pollution on the Inhabitants around the Shiranui Sea, Japan. *Toxics* 2018, *6*, 39

**DOI:** 10.3390/toxics7020022

**Published:** 2019-04-11

**Authors:** Shigeru Takaoka, Tadashi Fujino, Yoshinobu Kawakami, Shin-ichi Shigeoka, Takashi Yorifuji

**Affiliations:** 1 Kyoritsu Neurology and Rehabilitation Clinic, 2-2-28 Sakurai-cho, Minamata 867-0045, Japan; 2 Kikuyou Hospital, 5587 Haramizu, Kikuyou 869-1102, Japan; tds-fujino@jcom.zaq.ne.jp; 3 Minamata Kyoritsu Hospital, 2-2-12 Sakurai-cho, Minamata 867-0045, Japan; mkkawa@fsinet.or.jp (Y.K.); shigeoka@mk-kyouritu.com (S.-i.S.); 4 Department of Human Ecology, Graduate School of Environmental and Life Science, Okayama University, 3-1-1 Tsushima-naka, Kita-ku, Okayama 700-8530, Japan; yorichan@md.okayama-u.ac.jp

The authors wish to make the following corrections to this paper [1]:

1. Page 1, Abstract, lines 12–15: Period of time changed to match time between 1968 and the year in which our data was taken:

“Our data indicates that Minamata disease had spread outside of the central area and could still be observed recently, almost 50 years after the Chisso Company’s factory had halted the dumping of mercury polluted waste water back in 1968.” should be changed to “Our data indicates that Minamata disease had spread outside of the central area, and could still be observed recently, almost 40 years after the Chisso Company’s factory had halted the dumping of mercury polluted waste water back in 1968.”.

2. Page 3, paragraph 2, lines 5–7: Restructuring of sentence to make it clearer:

“Applicants seeking recognition were being socially discriminated against and the lack of a comprehensive pollution survey meant that many residents with health problems had not sought diagnosis.” should be changed to “Due to social discrimination and the lack of a comprehensive pollution survey, residents with health problems were reluctant to seek a diagnosis.”.

3. Page 4, paragraph 2, lines 2–4: Incorrect word replaced by correct one:

“We performed this survey in order to research the prevalence of signs and symptoms as well as the geometrical and chronological spread of health problems caused by methylmercury.” should be changed to “We performed this survey in order to research the prevalence of signs and symptoms, as well as the geographical and chronological spread of health problems caused by methylmercury.”.

4. Page 6, paragraph 1, lines 5–6: Missing word inserted:

“Those who had been born or had moved to the polluted on or after 1 January 1969 were classified under the third category (BA1968: *n* = 30, M/F = 21/9, Age = 37.4 ± 2.3).” should be changed to “Those who had been born or had moved to the polluted area on or after 1 January 1969 were classified under the third category (BA1968: *n* = 30, M/F = 21/9, Age = 37.4 ± 2.3).”, adding the word ‘area’.

5. Page 6, paragraph 2, lines 2–5: Rearrange order at the end of the paragraph:

“To evaluate this group, we selected 88 out of 227 subjects whose age was lower than 49 from the Control Area (M/F = 40/48, Age = 37.5 ± 6.0), and 84 out of 786 exposed subjects in the designated area who were born after 31 December 1968 (M/F = 44/40, Age = 44.8 ± 2.3) and whose age was lower than 49 from the four exposed groups.” should be changed to “To evaluate this group, we selected 88 out of 227 subjects, whose age was lower than 49, from the Control Area (M/F = 40/48, Age = 37.5 ± 6.0) and 84 out of 786 exposed subjects in the designated area, who were born after 31 December 1968 and whose age was lower than 49, from the four exposed groups (M/F = 44/40, Age = 44.8 ± 2.3).”.

6. Page 8, 2.6.2, lines 2–3: Added missing words to clarify better:

“To evaluate the severity of the neurological signs, we added (a) mark(s) to positive signs and symptoms, and we calculated the total score in the exposed four groups.” should be changed to “To evaluate the severity of the neurological signs and symptoms, we added (a) mark(s) to positive signs and symptoms, and we calculated the total score in the exposed four groups and the control group.”, adding ‘and symptoms’, as well as ‘and the control group’.

7. Page 21, Section 3.5.2, line 1: Remove “other”:

“The frequency of fish ingestion was closely related to the onset year of other symptoms (Table 13, Figure 9).” should be changed to “The frequency of fish ingestion was closely related to the onset year of the symptoms (Table 13, Figure 9).”.

8. Page 23, Table 14: Table title shortened:

“Table 14. Score of signs and onset of symptoms in each area.” should be changed to “Table 14. Score of signs and onset of symptoms.”.

9. Page 27, Figure 17: Changed “symptoms” to “signs”:

“Figure 17. Prevalence of neurological signs in subjects with and without sensory disturbance. Except for the prevalence of sensory disturbance, that of other symptoms was lower in subjects without sensory disturbance than in subjects with sensory disturbance but was generally higher than the Control Area. The prevalence patterns were similar in exposed subjects with and without sensory disturbance except for the prevalence of sensory disturbance.” should be changed to “Figure 17. Prevalence of neurological signs in subjects with and without sensory disturbance. Except for the prevalence of sensory disturbance, that of other signs was lower in subjects without sensory disturbance, than in subjects with sensory disturbance, but was generally higher than the Control Area. The prevalence patterns were similar in exposed subjects with and without sensory disturbance except for the prevalence of sensory disturbance.”.

10. Page 32, Figure 18, Missing word inserted:

“Figure 18. Prevalence of symptoms when comparing nine questions common to all five studies. The prevalence of all characteristic symptoms for Minamata disease was very. In this study, we used the prevalence of “sensory numbness in both hands” instead of “numbness of hands and feet.”” should be changed to “Figure 18. Prevalence of symptoms when comparing nine questions common to all five studies. The prevalence of all characteristic symptoms for Minamata disease was very high. In this study, we used the prevalence of “sensory numbness in both hands” instead of “numbness of hands and feet.””, thus inserting ‘high’ as in ‘very high’ in the first sentence.

11. Page 32, paragraph 2, lines 1–2: The age range data used for ADL comparison was 60–69. This resulted in a change for the value for bodily hygiene from 10.6% to 7.0%. The scientific results, however, remain the same:

“Although activities of daily living (ADL) of these patients decreased from 60 years old, 9.1% still needed assistance in eating, 11.6% in bodily hygiene and 10.6% in using the toilet [11].” should be changed to “Although activities of daily living (ADL) of these patients decreased from 60 years old, 9.1% still needed assistance in eating, 11.6% in bodily hygiene, and 7.0% in using the toilet in the age range of 60–69 [11].”, where the percentage of those using the toilet has been changed from 10.6% to 7.0%, and the words ‘in the age range of 60–69’ have also been added at the end.

12. Page 32, paragraph 3, lines 5–6: Referenced table numbers corrected:

“Tables 2 and 4, Figures 2 and 3 show that prevalence of specific symptoms as well as that of non-specific symptoms became higher through methylmercury exposure.” should be changed to “Tables 3 and 5, Figures 2 and 3 show that prevalence of specific symptoms as well as that of non-specific symptoms, became higher through methylmercury exposure.”. 

13. Page 33, 4.3, lines 7–8: The following sentence should be removed, as its meaning is incorrect:

This is the first study that dose-response effects were observed in methylmercury poisoning in Japan.

14. Page 34, 4.6, paragraph 3, lines 1–2: Reworded to clear up possible ambiguity:

“It is difficult to determine whether subjects of BA1968 (as displayed in Figure 14) had developed their symptoms due to continued exposure after 1968 or if they were late developing symptoms.” should be changed to “We can understand that subjects of BA1968 (as displayed in Figure 14) had developed their symptoms due to continued exposure after 1968. But it is impossible to determine whether subjects who were born before BA1968 and had developed their symptoms after 1968 had developed their symptoms due to continued exposure after 1968 or if they were late developing symptoms resulting from exposure before 1968.”.

15. Page 34, Section 4.7, lines 3–5 Added reference to some international studies:

“Outside of Japan, epidemiological studies have not seen such extreme neurological signs, but other more mild or latent neurocognitive and behavioral symptoms [23,24].” should be changed to “Outside of Japan, epidemiological studies with such extreme neurological signs are rare except for Iraq, Canada, and so on, but many other more mild or latent neurocognitive and behavioral symptoms have been reported [23,24].”.

16. Page 35, 4.8, paragraph 2, line 1: Reworded to clear up possible ambiguity:

“Secondly, the lifestyle and occupations were not the same among the four groups.” should be changed to “Secondly, the lifestyle and occupations were not the same between exposed and control groups.”.

17. Page 36, reference 12. Misspelling corrected:

“12. Sugiura, A. Health conditions among fisheren living in the Minamata disease prevalent area. *Jpn. J. Public Health* **1994**, *41*, 428–440. (In Japanese)” should be changed to “12. Sugiura, A. Health conditions among fishermen living in the Minamata disease prevalent area. *Jpn. J. Public Health* **1994**, *41*, 428–440. (In Japanese)”.

18. Page 36, reference 17. Incorrect reference to “Bristol, UK” removed:

“17. Central Council for Environmental Pollution Control. *About Future Measures to Minamata Disease*; The Environmental Agency: Bristol, UK, 1991. (In Japanese)” should be changed to “17. Central Council for Environmental Pollution Control. *About Future Measures to Minamata Disease*; The Environmental Agency: Tokyo, Japan, 1991. (In Japanese)”.

None of the above changes affect the scientific results. The manuscript will be updated and the original will remain online on the article webpage. We apologize for any inconvenience caused to our readers.
